# Hydrogenation of Terminal Alkenes Catalyzed by Air‐Stable Mn(I) Complexes Bearing an N‐Heterocyclic Carbene‐Based PCP Pincer Ligand

**DOI:** 10.1002/chem.202302455

**Published:** 2023-11-30

**Authors:** Daniel P. Zobernig, Michael Luxner, Berthold Stöger, Luis F. Veiros, Karl Kirchner

**Affiliations:** ^1^ Institute of Applied Synthetic Chemistry TU Wien Getreidemarkt 9/163-AC 1060 Wien Austria; ^2^ X-ray Center TU Wien Getreidemarkt 9/163 1060 Wien Austria; ^3^ Centro de Química Estrutural, Institute of Molecular Sciences Departamento de Engenharia Química Instituto Superior Técnico Universidade de Lisboa Av. Rovisco Pais 1049 001 Lisboa Portugal

**Keywords:** alkenes, DFT, hydrogenation, manganese, pincer complexes

## Abstract

Efficient hydrogenations of terminal alkenes with molecular hydrogen catalyzed by well‐defined bench stable Mn(I) complexes containing an N‐heterocyclic carbene‐based PCP pincer ligand are described. These reactions are environmentally benign and atom economic, implementing an inexpensive, earth abundant non‐precious metal catalyst. A range of aromatic and aliphatic alkenes were efficiently converted into alkanes in good to excellent yields. The hydrogenation proceeds at 100 °C with catalyst loadings of 0.25–0.5 mol %, 2.5–5 mol % base (KO^t^Bu) and a hydrogen pressure of 20 bar. Mechanistic insight into the catalytic reaction is provided by means of DFT calculations.

## Graphical abstract

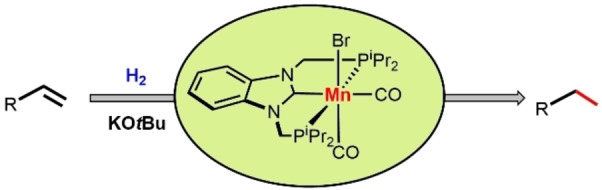

## Introduction

**Scheme 1:**
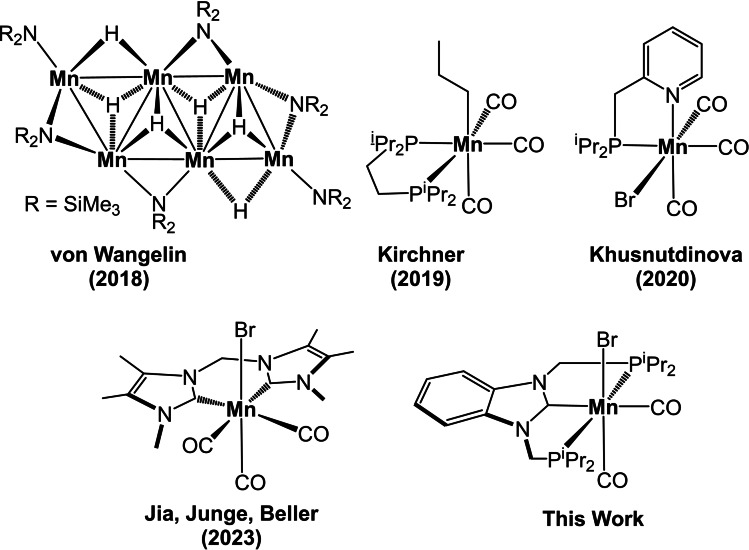
Manganese Catalysts for the Hydrogenation of Alkenes.

The development of efficient and selective synthetic methods to obtain novel target molecules is one of the fundamental research goals in modern organic chemistry. Moreover, as the sustainable use of resources is a major issue, it is important to perform organic reactions under catalytic conditions utilizing primarily earth abundant non‐precious metal catalysts.^[1]^ In particular, base metals such as iron and manganese are promising candidates as these belong to the most abundant metals in the Earth crust, are inexpensive, and exhibit a low environmental impact. In this context, of paramount importance are atom‐efficient and clean processes which permit access to valuable products such as alkanes, alcohols and amines via hydrogenation of olefins, alkynes, carbonyl compounds, and nitriles with molecular hydrogen.[^2^, ^3^] While iron, cobalt, and nickel hydrogenation catalysts had been subject of intense investigation over the past decade,[^4^, ^5^, ^6^, ^7^, ^8^, ^9^] low valent Mn(I) catalysts appeared in 2016 as new but very powerful players in this field.[^10^, ^11^] However, as the hydrogenation of unactivated C=C double bonds is concerned, examples of manganese catalysts are exceedingly rare.[^12^, ^13^, ^14^, ^15^] According to our knowledge, there are four known examples reported as shown in Scheme 1.

Inspired by recent discoveries of manganese catalyzed hydrogenation reactions.[^10^, ^11^, ^12^, ^13^, ^14^, ^15^] we describe here the efficient hydrogenation of terminal alkenes catalyzed by an air‐stable Mn(I) PCP pincer complex as shown in Scheme 1.

## Results and Discussion

**Scheme 2:**
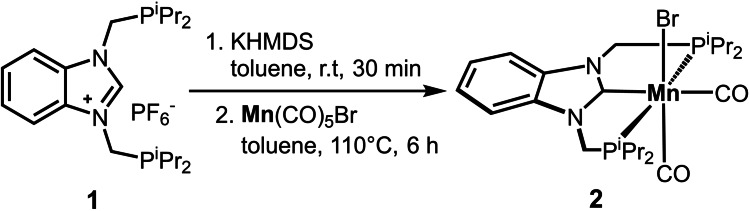
Synthesis of *cis*‐[Mn(PCP‐*i*Pr)(CO)_2_(Br)] (**2**).

Upon treatment of P(CH)P‐*i*Pr (**1**) with KHMDS in toluene and subsequent addition of [Mn(CO)_5_Br] while heating to 110 °C for 6 h, the neutral Mn(I) complex *cis*‐[Mn(PCP‐*i*Pr)(CO)_2_(Br)] (**2**) was obtained in 62 % isolated yield (Scheme 2). The resulting yellow complex was fully characterized by ^1^H, ^13^C{^1^H} and ^31^P{^1^H} NMR and IR spectroscopy, high‐resolution mass spectrometry and X‐ray crystallography (see Supporting Information). The IR spectrum contains two strong CO stretching vibrations at 1916 and 1835 cm^−1^ which clearly indicates the coordination of two CO ligands to the metal center in a *cis*‐arrangement.

This complex exhibits similar spectroscopic and metric properties than related Mn(I) complexes featuring NHC ligands recently described by Canac and co‐workers.^[16]^

The catalytic performance of complex **2** was then investigated for the hydrogenation of 4‐chlorostyrene as model substrate. We tested the influence of various solvents, concentration of base, pressures, and temperatures. Selected optimization experiments are depicted in Table 1. With a catalyst loading of 2 mol % at 100 °C in the presence of 10 mol % KO^t^Bu and a hydrogen pressure of 50 bar H_2_ in toluene after 18 h, 1‐chloro‐4‐ethylbenzene was obtained in essentially quantitative yield (Table 1, entry 1). No reaction took place in the protic solvent EtOH as well as MeCN, while in THF the conversion was slightly lower than in toluene (Table 1, entries 4–6). Lowering the catalyst loading to 0.5 mol % while keeping the concentration of the base at 10 mol % at 100 and 70 °C, respectively, led in the case of the latter to a significantly lower conversion (Table 1, entries 2 and 3). In the absence of base, no reaction took place. By lowering the catalyst loading to 0.25 mol % and the concentration of the base to 2.5 mol % at a hydrogen pressure of 20 bar high catalytic activity was observed and 4‐chlorostyrene was efficiently reduced to the corresponding alkane in >99 % yield (Table 1, entry 8). A further lowering of the base concentration from 2.5 to 2 and 1.5 mol % led to a decreased yield of 1‐chloro‐4‐ethylbenzene (Table 1, entries 9 and 10). It has to be noted that in the absence of catalyst no reaction took place. Moreover, the homogeneity of the system was confirmed upon addition of one drop of mercury, whereas no decrease of reactivity and selectivity was observed. If the catalytic reaction is performed in the presence of PEt_3_ no hydrogenation reaction took place indicating that PEt_3_ coordinates to the Mn(I) center blocking the vacant site for incoming substrates, even at a catalyst loading of 2 mol %.

**Table 1:** Optimization of the Reaction Conditions for the Hydrogenation of 4‐Chlorostyrene.^[a]^

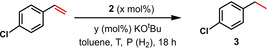						
entry	x [mol %]	y [mol %]	solvent	T [°C]	P [bar]	Yield [%]^[b]^
1	1	10	toluene	100	50	>99
2	0.5	10	toluene	100	50	82
3	0.5	10	toluene	70	50	14
4	0.5	10	THF	100	50	78
5	0.5	10	MeCN	100	50	n. d.
6	0.5	10	EtOH	100	50	n. d.
7	0.5	5	toluene	100	20	97
8	0.25	2.5	toluene	100	20	>99
9	0.25	2	toluene	100	20	93
10	0.25	1.5	toluene	100	20	71

[a] Reaction conditions: 4‐chlorostyrene (0.22 mmol), **2** (x mol %), KO^t^Bu (y mol %), 1 mL anhydrous solvent, 20–50 bar H_2_, 18 h. [b] Determined by GC MS.

Having established the best reaction conditions, the applicability of catalyst **2** is demonstrated in the selective hydrogenation of various aromatic and aliphatic alkenes. These results are shown in Table 2. In general, electron‐donating and electron‐withdrawing substituents, for example, chloride, bromide, methyl, methoxy and amino are well tolerated. The catalyst loading for a number of substrates was as low as 0.25 mol % while still maintaining excellent conversions (**3 a**–**3 f**, **3 r**). Styrene derivatives bearing bromide and amine moieties in the *para*‐ and *meta*‐position also exhibited very good conversions, albeit requiring a catalyst loading of 0.5 mol % (**3 g**–**3 j**). Steric hindrance significantly diminished the catalytic activity and lower yields were obtained for *ortho*‐substituted substrates such as electron‐rich 2,4,6‐trimethylstyrene (**3 k**) affording 43 % of alkane. N‐Vinylcarbazole, on the other hand, was reduced in essentially quantitative yield (**3 l**). Aliphatic substrates as well as substrates bearing phenyl and amine moieties could easily be hydrogenated in excellent yields (**3 m**–**3 t**). Allyl substrates showed only moderate conversions which might be due to isomerization of the substrates giving internal alkenes (**3 u**, **3 v**), which is a reactivity previously reported only once for manganese.^[17]^ Unfortunately, internal alkenes were not hydrogenated (**3 w**). Likewise, an ester‐functionalized styrene (**3 x**) showed no reactivity which may be due to side reactions involving the ester functionality. A control reaction with 4‐fluoroacetophenone (2 mol % **2**, 10 mol % KO^t^Bu,1 mL toluene, 20 bar H_2_, 18 h) was done with no conversion being observed.

**Table 2:** Hydrogenation of Various Aromatic and Aliphatic Alkenes Catalyzed by Complex **2**.^[a]^

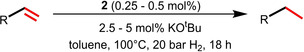
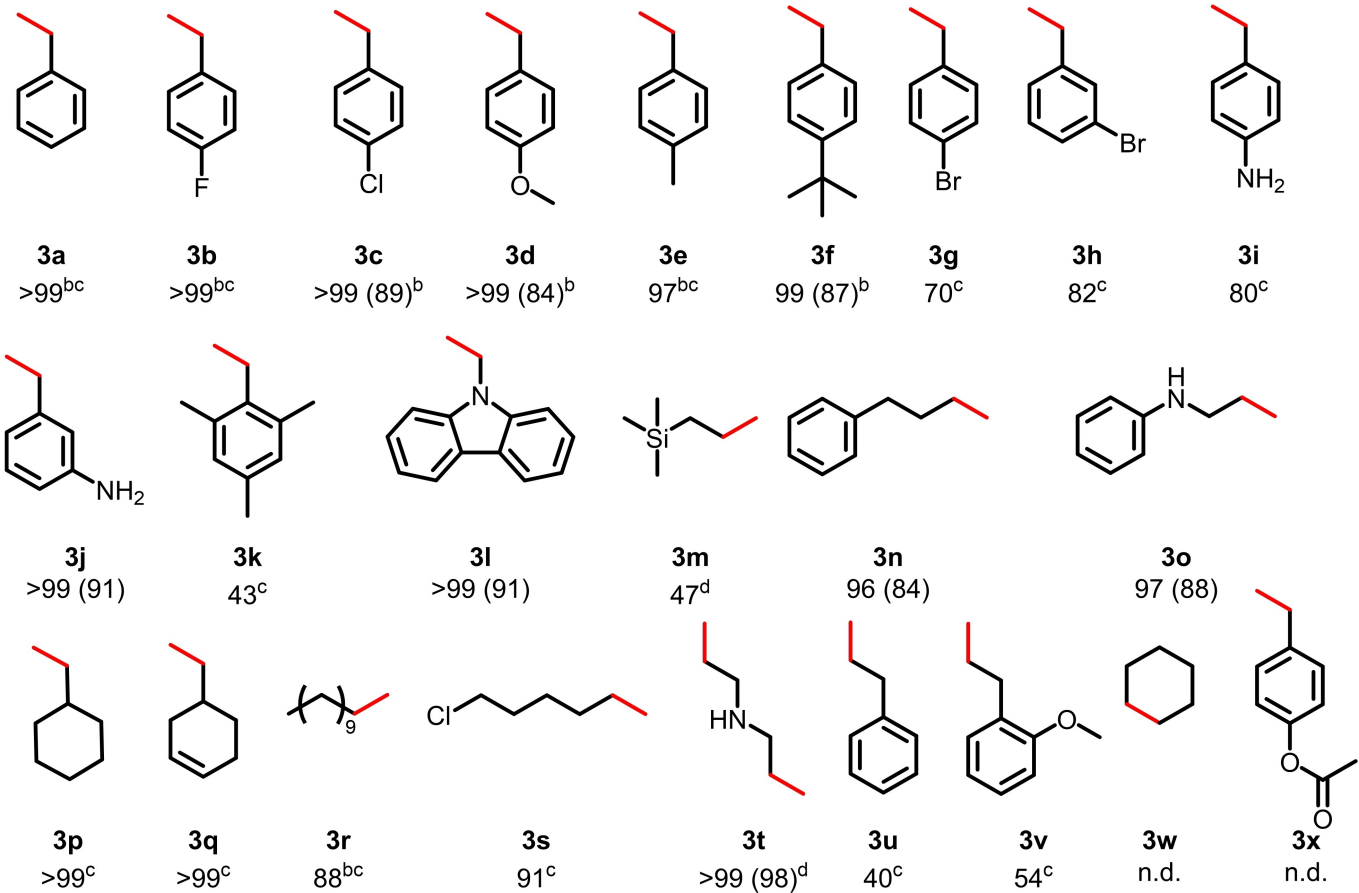

[a] Reaction conditions: alkene (0.88 mmol, 1 equiv), **2** (0.5 mol %), KO^t^Bu (5 mol %), 1 mL toluene, 100 °C, 20 bar H_2_, 18 h, conversion determined by GC‐MS, isolated yield in parentheses. [b] **2** (0.25 mol %), KO^t^Bu (2.5 mol %). [c] Yield determined by GC‐MS using *n*‐dodecane as standard. [d] Yield determined by ^1^H NMR spectroscopy with dibromomethane as standard.

**Scheme 3:**
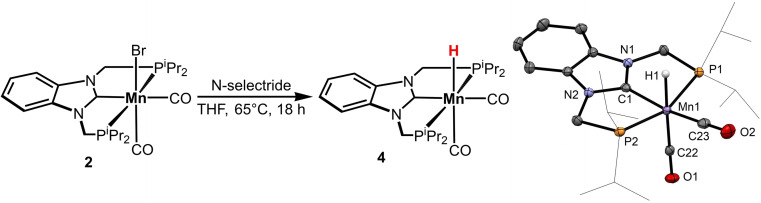
Synthesis of *cis*‐[Mn(PCP‐*i*Pr)(CO)_2_(H)] (**4**) and Structural View of **4** Showing 50 % Ellipsoids (Most H‐Atoms Omitted for Clarity). Selected bond distances (Å) and angles (deg): Mn1−P1 2.2386(7), Mn1−P2 2.2288(7), Mn1−C1 1.948(2), Mn1−C22 1.778(2), Mn1−C23 1.795(2), Mn1−H1 1.60(2), P1−Mn1−P2 159.23(2), C1−Mn1−H1 84.7(6), P1−Mn1−C23 97.83(6).

Monitoring the reaction by ^1^H NMR revealed that in the presence of H_2_ and base complex **2** is readily converted into the hydride complex *cis*‐[Mn(PCP‐*i*Pr)(CO)_2_(H)] (**4**). Mn hydrides are often proposed as reactive intermediates in hydrogenation reactions.[^14^, ^18^] Complex **4** was synthesized by reacting complex **2** with N‐selectride in THF at 65 °C for 18 h in 56 % isolated yield (Scheme 3). The ^1^H NMR spectrum of **4** displays a triplet centered at −8.37 ppm with a coupling constant ^*2*^ *J_PH_* of 46.1 Hz which agrees with the *cis*‐arrangement of the hydride and phosphine moieties. In the IR spectrum, two strong absorption bands at 1888 and 1828 cm^−1^ are observed which is characteristic of a *cis* arrangement of the two CO ligands. In comparison to the corresponding bromido complex these vibrations are found at 1916 and 1835 cm^−1^ in agreement with the higher electron donation of the hydrido ligand compared to the bromido ligand. The molecular structure of **4** was determined by single crystal X‐ray diffraction. A structural view with selected bond distances and angles is depicted in Scheme 3.

The catalytic activity of complex **4** (1 mol % **4**, 2 mol % KO^t^Bu, 1 mL toluene, 50 bar H_2_, 18 h, >99 % conversion) was also tested revealing that this compound exhibits similar reactivities for the hydrogenation of alkenes in the presence of base than complex **2**. Accordingly, the Mn hydride **4** seems to be an intermediate in alkene hydrogenation. It is interesting to note, in the absence of base **4** is catalytically inactive suggesting that another deprotonation step is required.

A plausible catalytic cycle based on experimental data and DFT calculations^[28]^ (see Supporting Information.) with styrene as model substrate and *cis*‐[Mn(PCP‐*i*Pr)(CO)_2_(H)] (**4**) as pre‐catalyst was established.

**Scheme 4:**
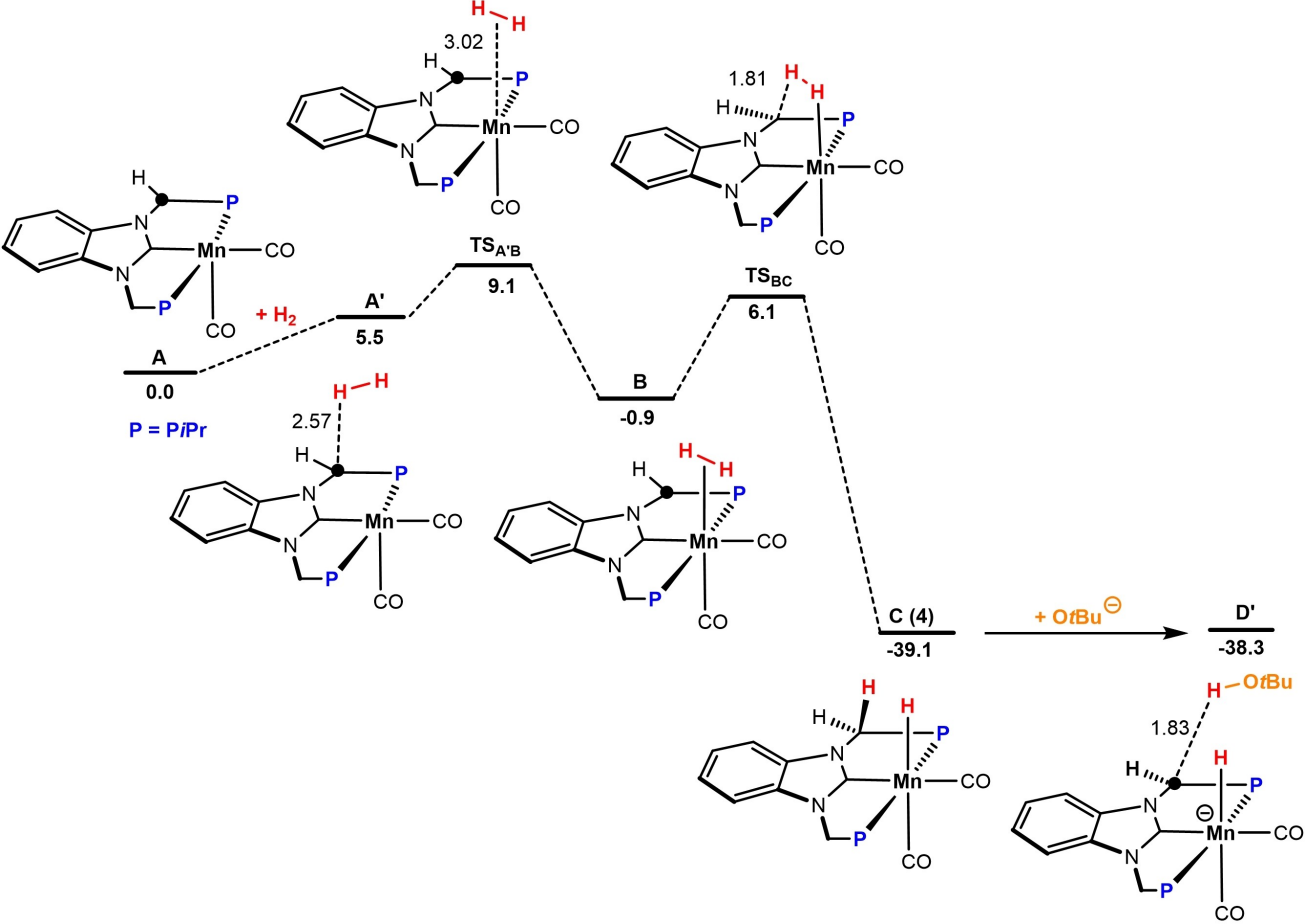
Free‐energy Profile for the Activation of Dihydrogen Involving Metal‐Ligand Cooperation and Deprotonation to Yield the Catalytically Active Species **D** (**D’** with weakly bound *t*BuOH). Free energies (kcal/mol) are referred to **A**. The C‐atom marked with a circle is formally negatively charged.

**Scheme 5:**
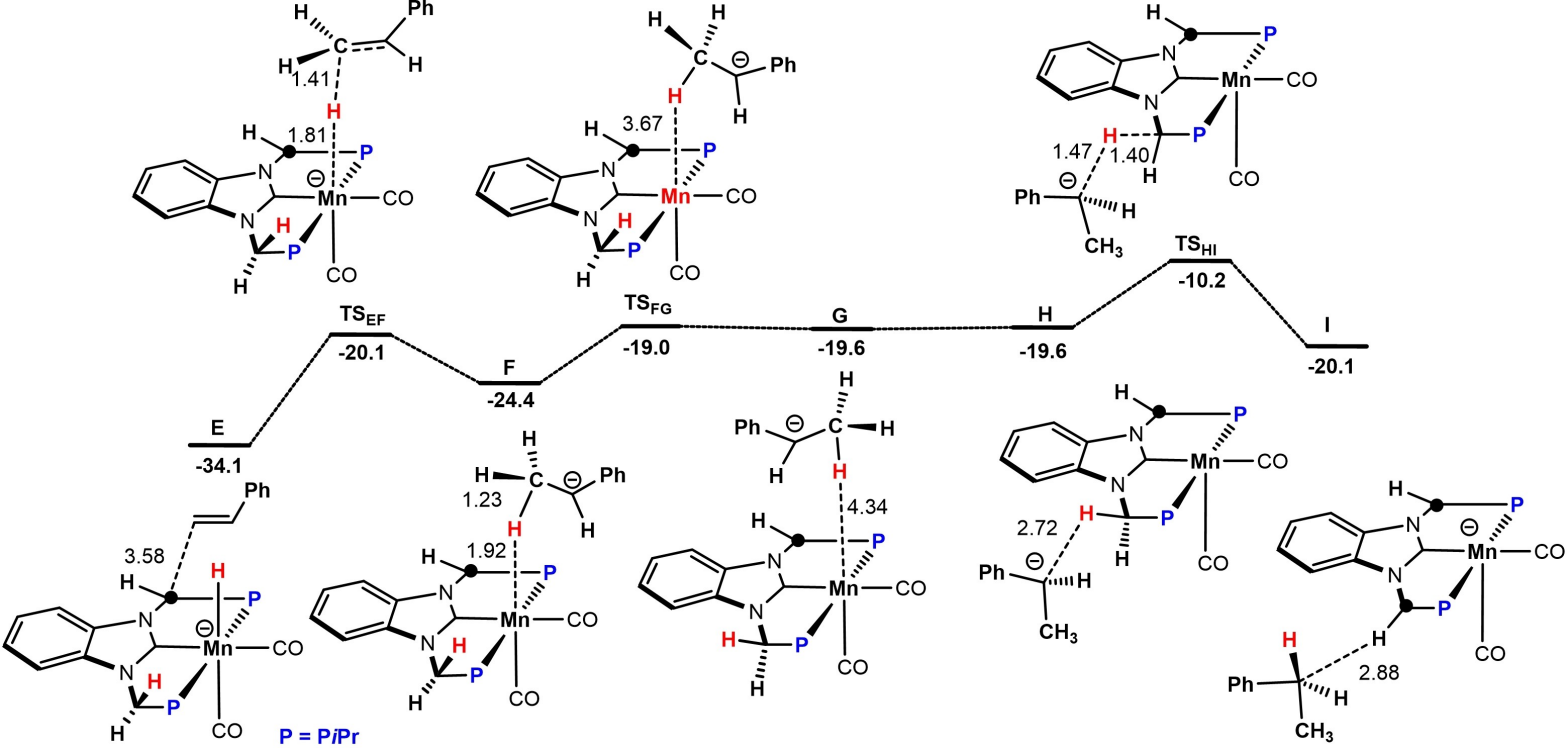
Free‐energy Profile for the Hydrogenation of Styrene via an Outer‐Sphere Pathway. Free energies (kcal/mol) are referred to **A**. The C‐atom marked with a circle is formally negatively charged.

**Scheme 6:**
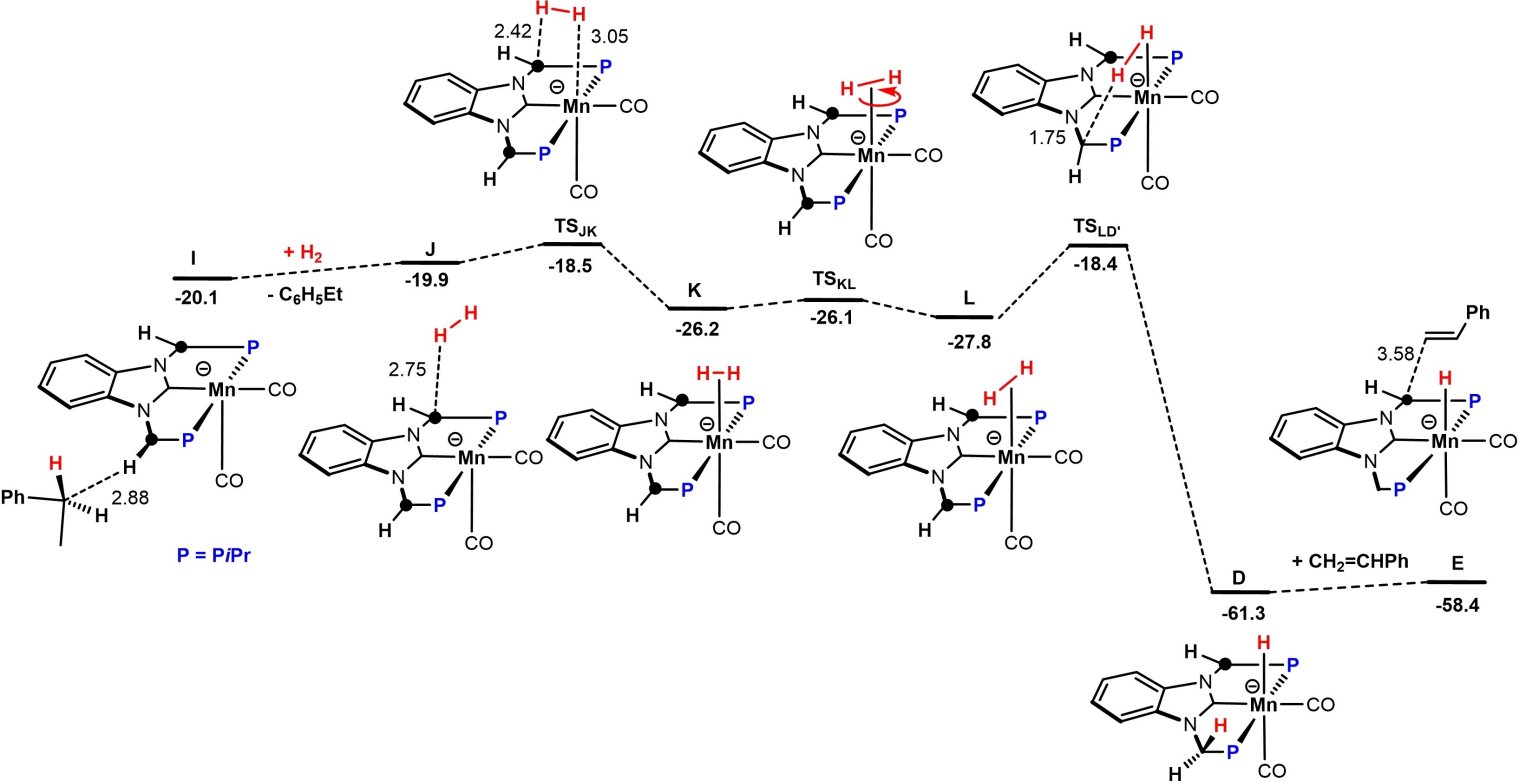
Free‐energy Profile for the Activation of Dihydrogen Involving Metal‐Ligand Cooperation reforming the Catalytically active Species **D**. Free energies (kcal/mol) are referred to **A**. The C‐atom marked with a circle is formally negatively charged.

**Scheme 7:**
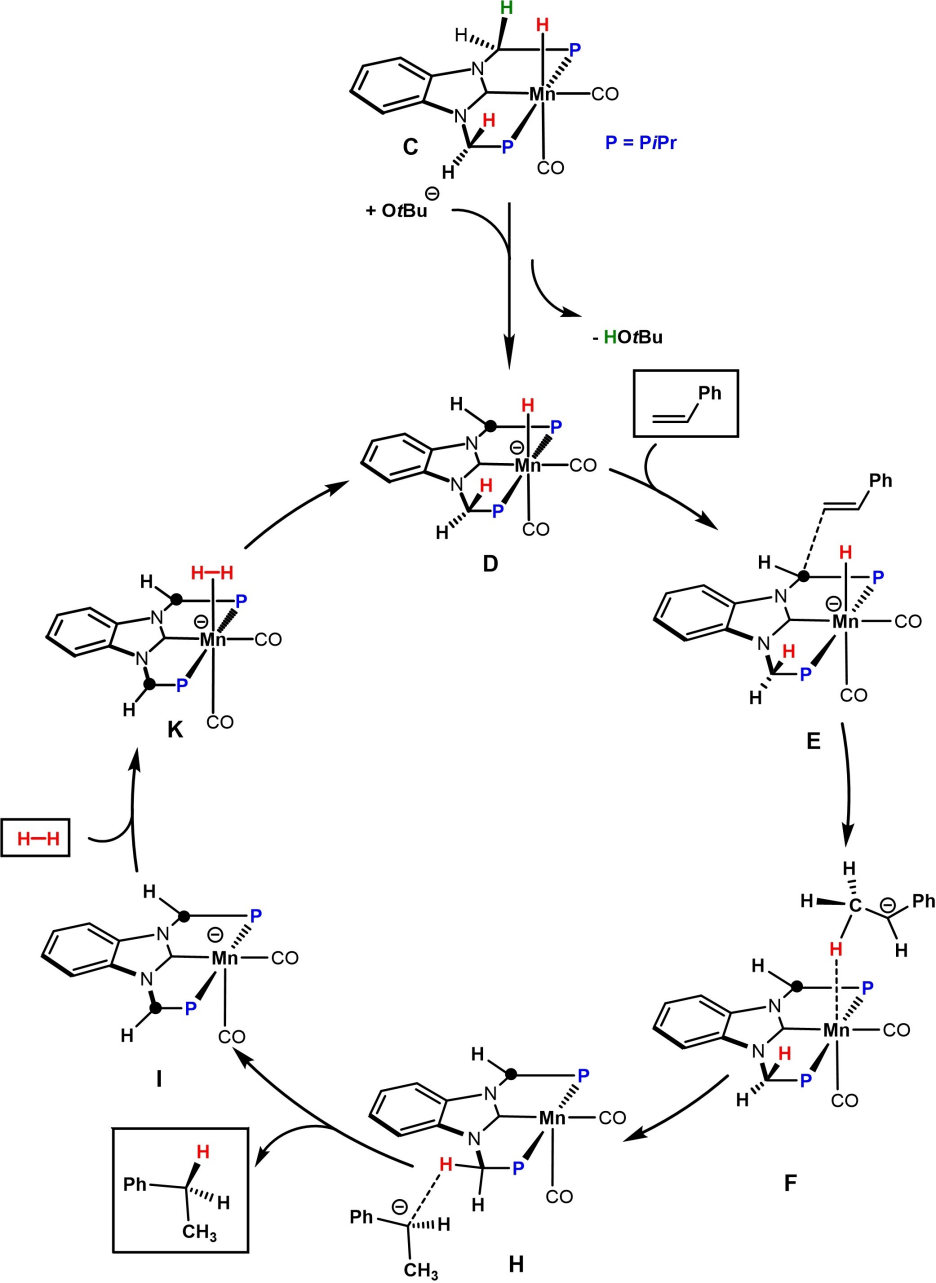
Simplified Catalytic Cycle for the Hydrogenation of Styrene.

The 16e^−^ intermediate **A** is formed after deprotonation of the methylene linker of the PCP ligand and removal of bromide from **2** in the presence of KO^*t*^Bu (Scheme 4). H_2_ coordination affords the hydrogen complex **B**. Heterolytic H_2_ splitting via a metal‐ligand cooperative pathway leads then to the formation of the Mn hydride complex **4** (**C** in the calculations) and re‐protonation of the methylene linker. Despite several efforts, complex **A** could neither be isolated nor detected spectroscopically. The methylene linker of complex **4** is readily deprotonated in the presence of KO*t*Bu to afford the anionic hydride complex **D** (**D’** with weakly bound *t*BuOH) which is the catalytically active species (see Supporting Information, Figure S2). It has to be noted an anionic Mn(I) hydride species was reported recently.^[19]^ In contrast to the neutral hydride complex **C**, complex **D** readily reacts with styrene in a formal nucleophilic attack of the hydride in **D** to the terminal carbon in styrene, from **E** to **F** (Scheme 5). This process is followed by a stepwise proton transfer to the internal carbon of styrene to form via intermediates **G** and **H** complex **I** where the second methylene linker is now also deprotonated. This outer‐sphere mechanism is similar to the ones proposed for manganese and iron catalyzed alkene hydrogenation by Khusnutdinova et al.^[14]^ and Jones and co‐workers, respectively.^[9c]^ The catalytically active species **D** is finally reformed via heterolytic cleavage of H_2_ via an bifunctional mechanism, i. e. metal ligand cooperation (Scheme 6). A simplified catalytic cycle is depicted in Scheme 7.

It has to be mentioned that phosphine arm dissociation or CO dissociation was considered as alternative pathways for pre‐catalyst activation, computational studies, however, ruled out these scenarios as the corresponding barriers were well above 40 kcal/mol.

## Conclusions

In sum, we have reported an efficient hydrogenation protocol of terminal alkenes with molecular hydrogen catalyzed by the well‐defined bench‐stable Mn(I) PCP pincer complex *cis*‐[Mn(PCP‐*i*Pr)(CO)_2_(Br)] (**2**) where metal‐ligand cooperation plays an important role as dihydrogen activation is concerned to form the hydride pre‐catalyst *cis*‐[Mn(PCP‐*i*Pr)(CO)_2_(H)] (**4**). These reactions are environmentally benign and atom economic implementing an inexpensive, earth abundant non‐precious metal catalyst. A range of aromatic and aliphatic alkenes were efficiently converted into alkanes in good to excellent isolated yields. The hydrogenation of proceeds at 100 °C with a catalyst loading of as low as 0.25 mol % and 2.5 mol % base (KO^t^Bu). A hydrogen pressure of 20 bar was applied and the reaction time was 18 h. Mechanistic insight into the catalytic reaction is provided by means of DFT calculations. The active catalyst is an anionic Mn(I) hydride complex which is formed upon deprotonation of one CH_2_ linker of *cis*‐[Mn(PCP‐*i*Pr)(CO)_2_(H)] (**4**). Olefin hydrogenation follows an *outer‐sphere* pathway via a stepwise hydride transfer and then proton transfer mechanism. Hydrogen activation to reform the active catalyst occurs via an *inner‐sphere* pathway involving metal‐ligand cooperation.

## Experimental Section

**General Information**. All reactions were performed under an inert atmosphere of argon by using Schlenk techniques or in a MBraun inert‐gas glovebox. The solvents were purified according to standard procedures.^[20]^ All alkene‐substrates were purchased from Sigma‐Aldrich, Acros Organics or TCI and used as purchased without further purification. The deuterated solvents were purchased from Eurisotope and dried over 3 Å molecular sieves.^1^H, ^13^C{^1^H} and ^31^P{^1^H} NMR spectra were recorded on Bruker AVANCE‐250, AVANCE‐400, and AVANCE‐600 spectrometers. ^1^H and ^13^C{^1^H} NMR spectra were referenced internally to residual protio‐solvent, and solvent resonances, respectively, and are reported relative to tetramethylsilane (δ=0 ppm). ^31^P{^1^H} NMR spectra were referenced externally to H_3_PO_4_ (85 %) (δ=0 ppm). Preparative flash column chromatography was conducted manually using glass columns packed with silica gel 60 (Merck, 40–63 μm).

High resolution‐accurate mass data mass spectra were recorded on a hybrid Maxis Qq‐aoTOF mass spectrometer (Bruker Daltonics, Bremen, Germany) fitted with an ESI‐source. Measured accurate mass data of the [M]^+^ ions for confirming calculated elemental compositions were typically within ±5 ppm accuracy. The mass calibration was done with a commercial mixture of perfluorinated trialkyl‐triazines (ES Tuning Mix, Agilent Technologies, Santa Clara, CA, USA).

GC‐MS analyses were conducted on a ISQ LT Single quadrupole MS (Thermo Fisher) directly interfaced to a TRACE 1300 Gas Chromatographic systems (Thermo Fisher), using a Rxi‐5Sil MS (30 m, 0.25 mm ID) cross‐bonded dimethyl polysiloxane capillary column at a carrier flow of He 1.5 mL/min. The oven program temperature was: Method **A**: 100 °C (2 min)//35 °C/min//300 °C (4 min). Method **B**: 40 °C (2.5 min)//12 °C/min//220 °C (2.5 min). If not stated otherwise, Method **A** was used as default.

Deposition Numbers 2278843 (for 2) and 2278844 (for 4) contains the supplementary crystallographic data for this paper. These data are provided free of charge by the joint Cambridge Crystallographic Data Centre and Fachinformationszentrum Karlsruhe Access Structures service.

***cis*** **‐[Mn(PCP)(CO)_2_Br] (2)**: A suspension of complex **1** (250 mg, 477 μmol, 1.0 equiv.) and KHMDS (105 mg, 524 μmol, 1.1 equiv.) in toluene (10 mL) was stirred for 30 min at room temperature. The reaction mixture was then heated to 110 °C and [Mn(CO)_5_Br] (131 mg, 477 μmol, 1.0 equiv.) was added and stirred for 6 h at that temperature. The yellow suspension was then filtrated through a syringe filter and the solvent was removed under reduced pressure. The yellow solid was then washed with *n*‐pentane (2×10 mL) yielding 167 mg (62 %) of **2**. Crystals for X‐ray diffraction were obtained by slow evaporation of a saturated toluene solution. ^1^H NMR (600 MHz, CD_2_Cl_2_): δ=7.41–7.37 (m, 2H), 7.34–7.29 (m, 2H), 4.47–4.34 (m, 4H), 3.14–3.05 (m, 2H), 2.69–2.60 (m, 2H), 1.48–1.36 (m, 18H), 1.26 (q, J=7.0 Hz) ppm. ^31^P{^1^H} NMR (243 MHz, CD_2_Cl_2_): δ=106.3 (s) ppm. ^13^C{^1^H} NMR (151 MHz, CD_2_Cl_2_): δ=230.7, 229.7, 225.3, 135.9, 128.1 (t, *J*=24.2 Hz), 123.2, 110.8, 49.2–49.0 (m), 26.8 (t, *J*=7.1 Hz), 26.7 (t, *J*=9.2 Hz), 20.1, 19.9, 19.2, 19.0 ppm. FTIR (cm^−1^): 1916 (CO), 1835 (CO) HR‐MS: *m*/*z* calcd for C_23_H_37_BrMnN_2_O_2_P_2_ [M−Br]^+^ 489.1633, found 489.1634.

***cis*** **‐[Mn(PCP‐*i*Pr)(CO)_2_H] (4)**: A solution of complex **2** (50 mg, 88 μmol, 1.0 equiv.) and N‐selectride (1 M in THF, 264 μL, 264 μmol, 3.0 equiv.) in THF (6 mL) was heated to 65 °C for 18 h while continuously stirring. The solvent was removed in *vacuo* and the resulting solid was then extracted with toluene (2×4 mL). The combined organic phases were then taken to dryness and the complex was washed with *n*‐pentane (2×4 mL) yielding 24 mg (56 %) of **4** as a beige solid. Crystals for X‐ray diffraction were obtained by slow diffusion of *n*‐pentane into a saturated toluene solution. ^1^H NMR (600 MHz, C_6_D_6_): δ=7.00–6.94 (m, 2H), 6.70–6.64 (m, 2H), 3.52–3.42 (m, 4H), 2.27–2.17 (m, 2H), 2.02–1.92 (m, 2H), 1.42–1.35 (m, 6H), 1.25–1.16 (m, 12H), 0.93–0.86 (m, 6H), −8.37 (t, J=46.1 Hz, 1H) ppm. ^31^P{^1^H} NMR (243 MHz, C_6_D_6_): δ=132.6 ppm. ^13^C{^1^H} NMR (151 MHz, C6D6): δ=234.8, 232.4, 229.4, 135.8, 121.5, 109.2, 47.9–47.7 (m), 30.4 (t, J=7.0 Hz), 27.5 (t, J=9.8 Hz), 19.5, 19.1, 18.9, 18.7 ppm. FTIR (cm^−1^): 1888 (CO), 1828 (CO) HR‐MS: *m*/*z* calcd for C_23_H_37_BrMnN_2_O_2_P_2_ [M−H]^+^ 489.1633, found 489.1628.

**General Procedure for the Catalytic Reactions**: Inside an argon flushed glovebox, a screw cap vial (8 mL) was charged with catalyst (0.25–0.5 mol %), substrate (0.88 mmol, 1 equiv.), base (1.5–10 mol %) and dry solvent (1 mL). A stirring‐bar was added, the vial was closed, transferred out of the glovebox and was flushed multiple times with hydrogen gas in an autoclave. It was subsequently put under hydrogen gas at given pressure while stirring for 18 h at given temperature. Afterwards the reaction mixture was allowed to reach room temperature, depressurized and exposed to air to quench the catalyst. 2 μL of the sample was analyzed via GC‐MS. After exposing the sample to air for 1 h, the solution was filtered through a thin pad of *silica* with dichloromethane. The solvent was then carefully removed in *vacuo* to yield the pure product, which was characterized by ^1^H‐ and ^13^C{^1^H} NMR spectroscopy. If purification was not possible, the yield was determined by GC‐MS with *n*‐dodecane as standard. In some cases, the yield was determined by ^1^H NMR spectroscopy with dibromomethane as standard.

**X‐ray Crystallography**: X‐ray diffraction data of **2** (CSD 2278843) were collected at *T*=300 K in a dry stream of nitrogen on a STOE STADIVARI diffractometer system equipped with a Dectris Eiger CdTe hybrid photon counting detector using Cu‐*K*α radiation (λ=1.54186 Å). Data were reduced with X‐Area and an absorption correction was applied with the multi‐scan approach implemented in LANA.^[21]^ X‐ray diffraction data of **4** ⋅ (C_6_H_6_)_0.298_ ⋅ (C_5_H_12_)_0.202_ (CSD 2278844) were collected at *T*=100 K in a dry stream of nitrogen on a Bruker Kappa APEX II diffractometer system using Mo‐*K*α radiation (λ=0.71073 Å). Data were reduced to intensity values with SAINT and an absorption correction was applied with the multi‐scan approach implemented in SADABS.^[22]^ The crystal structures were solved by the dual‐space approach implemented in SHELXT^[23]^ and refined against *F* ^2^ with SHELXL.^[24]^ Non‐H atoms were refined with anisotropic atomic displacement parameters. H atoms connected to C were placed in calculated positions and refined as riding on the parent atom. The hydride H was refined freely. Molecular graphics were generated with the program MERCURY.^[25]^

**DFT Calculations**: Calculations were performed using the gaussian 09 software package^[26]^ and the PBE0 functional, without symmetry constraints. That functional uses a hybrid generalized gradient approximation (GGA), including 25 % mixture of Hartree–Fock^[27]^ exchange with DFT^[28]^ exchange‐correlation, given by Perdew, Burke and Ernzerhof functional (PBE).^[29]^ The optimized geometries were obtained with the Stuttgart Effective Core Potentials and associated basis set (SDD)^[30]^ for Mn, and a standard 6‐31G(d,p)^[31]^ for the remaining elements (basis b1). Transition state optimizations were performed with the Synchronous Transit‐Guided Quasi‐Newton Method (STQN) developed by Schlegel et al.,^[32]^ following extensive searches of the Potential Energy Surface. Frequency calculations were performed to confirm the nature of the stationary points, yielding one imaginary frequency for the transition states and none for the minima. Each transition state was further confirmed by following its vibrational mode downhill on both sides and obtaining the minima presented on the energy profile. The electronic energies (E_b1_) obtained at the PBE0/b1 level of theory were converted to free energy at 298.15 K and 1 atm (G_b1_) by using zero point energy and thermal energy corrections based on structural and vibration frequency data calculated at the same level.

Single point energy calculations were performed on the geometries obtained at the PBE0/b1 level using the same functional and a 6‐311++G(d,p) basis set.^[33]^ Solvent effects (toluene) were accounted for in all calculations (including geometry optimizations) by means of the Polarisable Continuum Model (PCM) initially devised by Tomasi and coworkers^[34]^ with radii and non‐electrostatic terms of the SMD solvation model, developed by Truhler et al.^[35]^ The free energy values presented (G_b2_‐D3) were corrected for dispersion by means of Grimme DFT‐D3 method^[36]^ with Becke and Johnson short distance damping,^[37]^ being derived from the electronic energy values obtained at the PBE0‐D3/6‐311++G(d,p)//PBE0/b1 level (E_b2_‐D3) according to the following expression: (G_b2_‐D3)=(E_b2_‐D3)+G_b1_‐E_b1_.

## Conflict of interest

The authors declare no conflict of interest.

1

## Supporting information

As a service to our authors and readers, this journal provides supporting information supplied by the authors. Such materials are peer reviewed and may be re‐organized for online delivery, but are not copy‐edited or typeset. Technical support issues arising from supporting information (other than missing files) should be addressed to the authors.

Supporting Information

## Data Availability

In the supplementary material of this article synthetic procedures and NMR spectra of ligand precursors, P(CH)P‐*i*Pr (**1**) and all organic compounds as well as cartesian coordinates for DFT‐optimized structures (XYZ files) are provided.
