# Supplementary material for: Hydrogenation of Terminal Alkenes Catalyzed by Air‐Stable Mn(I) Complexes Bearing an N‐Heterocyclic Carbene‐Based PCP Pincer Ligand
Source: Chemistry. 2023 Nov 30;30(4):e202302455. doi: 10.1002/chem.202302455 (PMC10952557; doi:10.1002/chem.202302455)
Supplement: Supplementary file 1 — Supporting Information [file CHEM-30-0-s001.pdf]

Chemistry–A European Journal

Supporting Information

Hydrogenation of Terminal Alkenes Catalyzed by Air-Stable Mn(I) Complexes Bearing an N-Heterocyclic Carbene-Based PCP Pincer Ligand

Daniel P. Zobernig, Michael Luxner, Berthold Stöger, Luis F. Veiros, and Karl Kirchner*

1 Syntheses S2

2 X-Ray Analysis..S3

3 DFT Calculations..S3

4 Mechanistic Studies S3

4.1 Poisoning with Triethylphosphine S3
4.2 Poisoning with Mercury..S4
4.3 Control Experiment without Catalyst..S4
4.4 Control Experiment with Hydride..S4
4.5 Control Experiment with Hydride without Base......................................S4
4.6 Hydrogenation of Ketones S4

5 Characterization of Organic Products...S5

6 References S9

7 NMR and IR Spectra S10

7.1 Ligand Spectra...S10
7.2 Complex Spectra S14
7.3 Spectra of Organic Products..S19

1 Syntheses

N^1,N^2-Bis[[bis(*i*-propyl)phosphinoselenoyl]methyl]-1,2-benzenediamine (5).

5 was synthesized using a modified procedure from literature.[1]

Paraformaldehyde (241 mg, 8 mmol, 1 equiv.) and diisopropylphosphine (948 mg, 8 mmol, 1 equiv.) were put in a Schlenk flask that was flushed with argon beforehand. The reaction mixture was heated to 60°C and stirred for 18 hours. The resulting oil and was then left to cool to room temperature, after which dry dichloromethane (20 mL) was added. *o*-Phenylenediamine (389 mg, 3.6 mmol, 0.45 equiv.) was added, and the solution was stirred for 48 hours at ambient temperature. Black selenium (695 mg, 8.8 mmol, 1.1 equiv.) was slowly added and the mixture was stirred for 1 hour. The suspension was then filtered off over a patch of celite. The residue was washed vigorously with dichloromethane after which the organic phase was taken to dryness. The product was purified *via* silica gel chromatography (eluent: dichloromethane), yielding 1301 mg (62%) of **5** as a yellow solid. ^{1}H NMR (400 MHz, CD_2Cl_2): δ = 6.84 – 6.78 (m, 2H), 6.72 – 6.67 (m, 2H), 4.25 – 4.15 (m, 2H), 3.41 (t, J = 6.4 Hz, 4H), 2.47 – 2.30 (m, 4H), 1.30 – 1.22 (m, 24H) ppm. ^{31}P {^{1}H} NMR (162 MHz, CD_2Cl_2): δ = 64.5 ppm. ^{13}C {^{1}H} NMR (101 MHz, CD_2Cl_2): δ = 137.9 (d, *J* = 11.4 Hz), 120.4, 112.4, 38.9 (d, *J* = 46.4 Hz), 27.3 (d, *J* = 41.4 Hz), 17.5 (dd, *J* = 19.3, 2.2 Hz) ppm. HR-MS: *m*/*z* calcd for $C_{20}H_{38}N_2NaP_2Se_2$ $[M+Na]^+$ 551.0732, found 551.0739.

1,3-Bis[[bis(*i*-propyl)phosphinoselenoyl]methyl]-1*H*-benzimidazolium hexafluorophosphate (6).

6 was synthesized using a modified procedure from literature.[1]

5 (1053 mg, 2 mmol, 1 eq.), triethyl orthoformate (1778 mg, 12 mmol, 6 equiv.) and ammonium hexafluorophosphate (326 mg, 2 mmol, 1 equiv.) were put in 1,2-dichloroethane (20 mL) under an argon atmosphere. The reaction mixture was heated to 80°C while stirring for 2 hours. The red solution was then taken to dryness, yielding a red solid. The solid was washed with cold methanol (2x10 mL) and with diethyl ether (3x10 mL), after which it was dried in *vacuo* to yield 1297 mg (96%) of **6** as a white solid. ^{1}H NMR (400 MHz, CD_2Cl_2): δ = 9.75 (s, 1H), 7.93 – 7.84 (m, 2H), 7.79 – 7.71 (m, 2H), 4.97 (d, *J* = 3.7 Hz, 4H), 2.54 – 2.36 (m, 4H), 1.33 – 1.19 (m, 24H) ppm. ^{31}P{^{1}H} NMR (162 MHz, CD_2Cl_2): δ = 67.7 (s), -144.4 (sept, J = 710 Hz) ppm. ^{13}C{^{1}H} NMR (101 MHz, CD_2Cl_2): δ = 141.3, 131.6, 128.3, 113.8, 42.6 (d, *J* = 31.8 Hz), 27.8 (d, *J* = 39.2 Hz), 17.2 (d, *J* = 38.9 Hz) ppm. HR-MS: *m*/*z* calcd for $C_{21}H_{37}F_6N_2P_3Se_2$ $[M]^+$ 539.0756, found 539.0764.

1,3-Bis[[bis(*i*-propyl)phosphino]methyl]-1*H*-benzimidazolium hexafluorophosphate (P(CH)P-*i*Pr) (1).

1 was synthesized using a modified procedure from literature.[2]

6 (682 mg, 1.0 mmol, 1 equiv.) and dry dichloromethane (20 mL) were put in a Schlenk flask that was flushed with argon beforehand. Tris(dimethylamino)phosphine (343 mg, 2.1 mmol, 2.1 equiv.) was added to the suspension and it was stirred for 1 hour. The resulting solution was concentrated in *vacuo* after which dry diethyl ether was added until all solid precipitated. The solvent was decanted off and the resulting white substance was washed with dry diethyl ether (4x10 mL). It was then dried in *vacuo* yielding 472 mg (90%) of **1** as a white powder. ^{1}H NMR (400 MHz, CD_2Cl_2): δ = 9.33 (s, 1H), 7.97 – 7.88 (m, 2H), 7.77 – 7.68 (m, 2H), 4.59 (d, *J* =

1.8 Hz, 5H), 2.08 – 1.93 (m, 4H), 1.29 – 0.99 (m, 24H) ppm. $^{31}P\{^{1}H\}$ NMR (162 MHz, CD_2Cl_2): δ = 5.6 (s), -144.4 (sept, J = 714 Hz) ppm. ^{13}C NMR (101 MHz, CD_2Cl_2): δ = 141.0, 132.4, 127.9, 114.5, 43.7 (d, *J* = 27.7 Hz), 23.6 (d, *J* = 12.6 Hz), 19.7 (d, *J* = 15.0 Hz), 18.9 (d, *J* = 9.8 Hz) ppm. HR-MS: *m/z* calcd for $C_{21}H_{37}F_6N_2P_3$ $[M]^+$ 379.2426, found 379.2436.

2 X-Ray Analysis

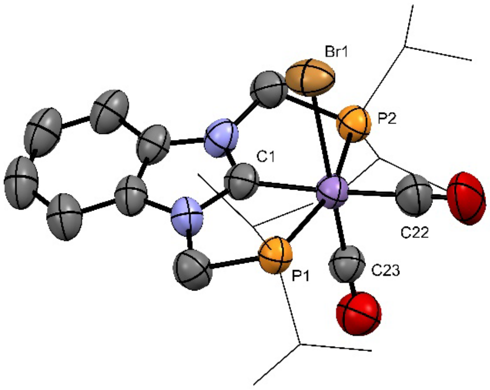

Figure S1. Structural view of **2** showing 50 % Ellipsoids (H Atoms Omitted for Clarity). Selected bond distances (Å) and angles (°): Mn1-P1 2.281(1), Mn1-P2 2.293(1), Mn1-C1 1.956(4), Mn1-C22 1.804(7), Mn1-C23 1.714(7), Mn1-Br1 2.605(1), P1-Mn1-P2 160.49, C1-Mn1-Br1 85.6(1), P1-Mn1-C23 86.6(2).

3 DFT Calculations

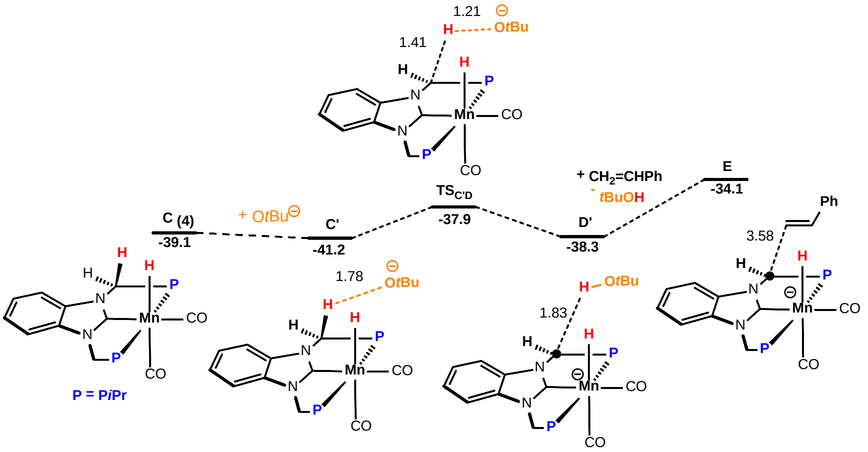

Figure S2. Free-energy Profile for the Deprotonation of **C** and Addition of Styrene. Free energies (kcal/mol) are referred to **A**. The C-atom marked with a circle is formally negatively charged.

4 Mechanistic Studies

4.1 Poisoning with Triethylphosphine

Inside an argon flushed glovebox, a screw cap vial (8 mL) was charged with 4-chlorostyrene (26 µL, 216 µmol, 1 equiv.), KO^{t}Bu (2.5 mg, 22 µmol, 10 mol%), **2** (2.5 mg, 4.3 µmol, 2.0 mol%), triethylphosphine (32 µL, 216 µmol, 1 equiv.) and dry toluene (1 mL) and closed under argon atmosphere. The vial was transferred out of the glovebox and was flushed multiple times with hydrogen gas in an autoclave. It was subsequently put under

hydrogen gas at 20 bar while stirring for 18 hours at 100°C. Afterwards the reaction mixture was allowed to reach room temperature, depressurized and exposed to air to quench the catalyst. The sample was analyzed *via* GC-MS. A conversion of only 9% could be observed.

4.2 Poisoning with Mercury

Inside an argon flushed glovebox, a screw cap vial (8 mL) was charged with 4-chlorostyrene (26 µL, 216 µmol, 1 equiv.), KO^{t}Bu (2.5 mg, 22 µmol, 10 mol%), **2** (2.5 mg, 4.3 µmol, 2.0 mol%), a drop of mercury and dry toluene (1 mL) and closed under argon atmosphere. The vial was transferred out of the glovebox and was flushed multiple times with hydrogen gas in an autoclave. It was subsequently put under hydrogen gas at 20 bar while stirring for 18 hours at 100°C. Afterwards the reaction mixture was allowed to reach room temperature, depressurized and exposed to air to quench the catalyst. The sample was analyzed *via* GC-MS. Full conversion was observed.

4.3 Control Experiment without Catalyst

Inside an argon flushed glovebox, a screw cap vial (8 mL) was charged with 4-chlorostyrene (26 µL, 216 µmol, 1 equiv.), KO^{t}Bu (2.5 mg, 22 µmol, 10 mol%) and dry toluene (1 mL) and closed under argon atmosphere. The vial was transferred out of the glovebox and was flushed multiple times with hydrogen gas in an autoclave. It was subsequently put under hydrogen gas at 20 bar while stirring for 18 hours at 100°C. Afterwards the reaction mixture was allowed to reach room temperature, depressurized and exposed to air. The sample was analyzed *via* GC-MS. No conversion was observed.

4.4 Control Experiment with Hydride

Inside an argon flushed glovebox, a screw cap vial (8 mL) was charged with 4-chlorostyrene (26 µL, 216 µmol, 1 equiv.), **4** (1.1 mg, 2.2 µmol, 1.0 mol%), KO^{t}Bu (0.5 mg, 4.4 µmol, 2.0 mol%) and dry toluene (1 mL) and closed under argon atmosphere. The vial was transferred out of the glovebox and was flushed multiple times with hydrogen gas in an autoclave. It was subsequently put under hydrogen gas at 50 bar while stirring for 18 hours at 100°C. Afterwards the reaction mixture was allowed to reach room temperature, depressurized and exposed to air. The sample was analyzed *via* GC-MS. Full conversion was observed.

4.5 Control Experiment with Hydride without Base

Inside an argon flushed glovebox, a screw cap vial (8 mL) was charged with 4-chlorostyrene (26 µL, 216 µmol, 1 equiv.), **4** (2.1 mg, 4.3 µmol, 2.0 mol%) and dry toluene (1 mL) and closed under argon atmosphere. The vial was transferred out of the glovebox and was flushed multiple times with hydrogen gas in an autoclave. It was subsequently put under hydrogen gas at 20 bar while stirring for 18 hours at 100°C. Afterwards the reaction mixture was allowed to reach room temperature, depressurized and exposed to air. The sample was analyzed *via* GC-MS. No conversion was observed.

4.6 Hydrogenation of Ketones

Inside an argon flushed glovebox, a screw cap vial (8 mL) was charged with 4-fluoroacetophenone (26 µL, 216 µmol, 1 equiv.), **2** (2.5 mg, 4.3 µmol, 2.0 mol%), KO^{t}Bu (2.5 mg, 22 µmol, 10 mol%), and dry toluene (1 mL) and closed under argon atmosphere. The vial was transferred out of the glovebox and was flushed multiple times with hydrogen gas in an autoclave. It was subsequently put under hydrogen gas at 20 bar while stirring for 18 hours at 100°C. Afterwards the reaction mixture was allowed to reach room temperature, depressurized and exposed to air. The sample was analyzed *via* GC-MS. No conversion was observed.

5 Characterization of Organic Products

Ethylbenzene (**3a**)

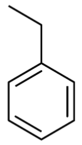

Styrene (101 µL, 0.88 mmol, 1 equiv.); catalyst (1.25 mg, 2.2 µmol, 0.25 mol%); KO^{t}Bu (2.5 mg, 22 µmol, 2.5 mol%); yield: 99% (according to GC-MS with *n*-dodecane as standard)

RT (GC): 5.37 min MS: 106.16 m/z $[M]^+$ (Method **B**)

1-Ethyl-4-fluorobenzene (**3b**)

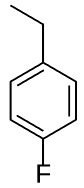

4-Fluorostyrene (106 µL, 0.88 mmol, 1 equiv.); catalyst (1.25 mg, 2.2 µmol, 0.25 mol%); KO^{t}Bu (2.5 mg, 22 µmol, 2.5 mol%); yield: 99% (according to GC-MS with *n*-dodecane as standard)

RT (GC): 5.64 min MS: 124.14 m/z $[M]^+$ (Method **B**)

1-Ethyl-4-chlorobenzene (**3c**)

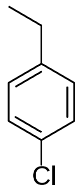

4-Chlorostyrene (113 µL, 0.88 mmol, 1 equiv.); catalyst (1.25 mg, 2.2 µmol, 0.25 mol%); KO^{t}Bu (2.5 mg, 22 µmol, 2.5 mol%); 110 mg (89%) of a colorless oil

^1H NMR (400 MHz, $CDCl_3$): δ = 7.28 – 7.19 (m, 2H), 7.14 – 7.06 (m, 2H), 2.60 (q, *J* = 7.6 Hz, 2H), 1.20 (t, *J* = 7.6 Hz, 3H) ppm.

$^{13}C\{^1H\}$ NMR (101 MHz, $CDCl_3$): δ 142.8, 131.4, 129.4, 128.5, 28.4, 15.7 ppm.

RT (GC): 3.03 min MS: 140.12 m/z $[M]^+$

This data corresponds with previously reported data.[3]

4-Ethylanisole (**3d**)

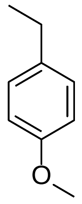

4-Vinylanisole (118 µL, 0.88 mmol, 1 equiv.); catalyst (1.25 mg, 2.2 µmol, 0.25 mol%); KO^{t}Bu (2.5 mg, 22 µmol, 2.5 mol; 101 mg (84%) of a colorless oil

^1H NMR (400 MHz, $CDCl_3$): δ = 7.16 – 7.08 (m, 2H), 6.88 – 6.80 (m, 2H), 3.79 (s, 3H), 2.60 (q, *J* = 7.6 Hz, 2H), 1.22 (t, *J* = 7.6 Hz, 3H) ppm.

$^{13}C\{^1H\}$ NMR (101 MHz, $CDCl_3$): δ = 157.8, 136.5, 128.8, 113.9, 55.4, 28.1, 16.0 ppm.

RT (GC): 3.39 min MS: 136.14 m/z $[M]^+$

This data corresponds with previously reported data.[4]

4-Ethyltoluene (**3e**)

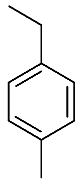

4-Methylstyrene (117 µL, 0.88 mmol, 1 equiv.); catalyst (1.25 mg, 2.2 µmol, 0.25 mol%); KO^{t}Bu (2.5 mg, 22 µmol, 2.5 mol%); yield: 97% (according to GC-MS with *n*-dodecane as standard)

RT (GC): 7.07 min MS: 120.17 m/z $[M]^+$ (Method **B**)

1-*tert*-Butyl-4-ethylbenzene (**3f**)

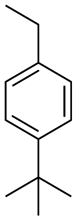

4-*tert*-Butylstyrene (162 µL, 0.88 mmol, 1 equiv.); catalyst (1.25 mg, 2.2 µmol, 0.25 mol%); KO^{t}Bu (2.5 mg, 22 µmol, 2.5 mol%); 126 mg (87 %) of a colorless oil

^{1}H NMR (400 MHz, $CDCl_3$): δ = 7.38 – 7.31 (m, 2H), 7.22 – 7.12 (m, 2H), 2.66 (q, *J* = 7.6 Hz, 2H), 1.35 (s, 9H), 1.27 (t, *J* = 7.6 Hz, 3H) ppm.

^{13}C{^{1}H} NMR (101 MHz, $CDCl_3$): δ = 148.5, 141.3, 127.7, 125.3, 34.5, 31.6, 28.4, 15.6 ppm.

RT (GC): 3.84 min MS: 162.20 m/z $[M]^+$

This data corresponds with previously reported data.[4]

1-Ethyl-4-bromobenzene (**3g**)

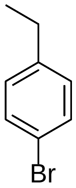

4-Bromostyrene (116 µL, 0.88 mmol, 1 equiv.); catalyst (2.5 mg, 4.3 µmol, 0.5 mol%); KO^{t}Bu (5 mg, 43 µmol, 5 mol%); yield: 70 % (according to GC-MS with *n*-dodecane as standard)

RT (GC): 3.62 min MS: 184.07 m/z $[M]^+$

1-Ethyl-3-bromobenzene (**3h**)

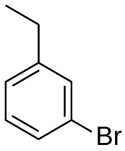

3-Bromostyrene (115 µL, 0.88 mmol, 1 equiv.); catalyst (2.5 mg, 4.3 µmol, 0.5 mol%); KO^{t}Bu (5 mg, 43 µmol, 5 mol%); yield: 82 % (according to GC-MS with *n*-dodecane as standard)

RT (GC): 3.58 min MS: 184.06 m/z $[M]^+$

4-Ethylaniline (**3i**)

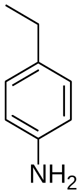

4-Vinylaniline (104 µL, 0.88 mmol, 1 equiv.); catalyst (2.5 mg, 4.3 µmol, 0.5 mol%); KO^{t}Bu (5 mg, 43 µmol, 5 mol%); yield: 80% (according to GC-MS with *n*-dodecane as standard)

RT (GC): 3.75 min MS: 121.14 m/z $[M]^+$

3-Ethylaniline (**3j**)

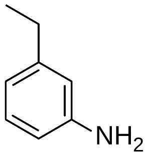

3-Vinylaniline (100 µL, 0.88 mmol, 1 equiv.); catalyst (2.5 mg, 4.3 µmol, 0.5 mol%); KO^{t}Bu (5 mg, 43 µmol, 5 mol%); 97 mg (91%) of a beige liquid

^{1}H NMR (400 MHz, $CDCl_3$): δ = 7.08 (t, *J* = 7.7 Hz, 1H), 6.66 – 6.59 (m, 1H), 6.58 – 6.49 (m, 2H), 3.61 (s, 2H), 2.57 (q, *J* = 7.6 Hz, 2H), 1.22 (t, *J* = 7.6 Hz, 3H) ppm.

^{13}C{^{1}H} NMR (101 MHz, $CDCl_3$): δ = 146.5, 145.7, 129.3, 118.4, 114.9, 112.6, 29.0, 15.6 ppm.

RT (GC): 3.76 min MS: 121.14 m/z $[M]^+$

This data corresponds with previously reported data.[4]

2-Ethyl-1,3,5-trimethylbenzene (**3k**)

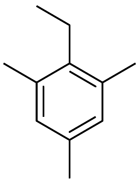

2,4,6-Trimethylstyrene (143 µL, 0.88 mmol, 1 equiv.); catalyst (2.5 mg, 4.3 µmol, 0.5 mol%); KO^{t}Bu (5 mg, 43 µmol, 5 mol%); yield: 43 % (according to GC-MS with *n*-dodecane as standard)

RT (GC): 3.86 min MS: 148.20 m/z $[M]^+$

N-Ethylcarbazole (**3l**)

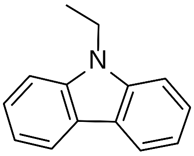

N-Vinylcarbazole (171 mg, 0.88 mmol, 1 equiv.); catalyst (2.5 mg, 4.3 µmol, 0.5 mol%); KO^{t}Bu (5 mg, 43 µmol, 5 mol%); 158 mg (91%) of a colorless solid

^{1}H NMR (400 MHz, $CDCl_3$): δ = 8.19 – 8.12 (m, 2H), 7.56 – 7.42 (m, 4H), 7.31 – 7.27 (m, 2H), 4.42 (q, *J* = 7.2 Hz, 2H), 1.48 (t, *J* = 7.2 Hz, 3H) ppm.

^{13}C{^{1}H} NMR (101 MHz, $CDCl_3$): δ = 140.1, 125.7, 123.1, 120.5, 118.9, 108.6, 37.6, 13.9 ppm.

RT (GC): 6.72 min MS: 195.16 m/z $[M]^+$

This data corresponds with previously reported data.[5]

Trimethylpropylsilane (**3m**)

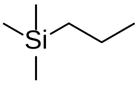

Allyltrimethylsilane (141 µL, 0.88 mmol, 1 equiv.); catalyst (2.5 mg, 4.3 µmol, 0.5 mol%); KO^{t}Bu (5 mg, 43 µmol, 5 mol%); yield: 47% (according to NMR with dibromomethane as standard)

Butylbenzene (**3n**)

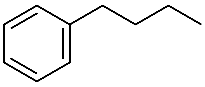

4-Phenyl-1-butene (133 µL, 0.88 mmol, 1 equiv.); catalyst (2.5 mg, 4.3 µmol, 0.5 mol%); KO^{t}Bu (5 mg, 43 µmol, 5 mol%); 100 mg (84%) of a colorless liquid

^{1}H NMR (400 MHz, $CDCl_3$): δ = 7.22 – 7.16 (m, 2H), 7.15 – 7.05 (m, 3H), 2.53 (t, *J* = 7.9 Hz, 2H), 1.58 – 1.47 (m, 2H), 1.35 – 1.22 (m, 2H), 0.85 (t, *J* = 7.3 Hz, 3H) ppm.

^{13}C{^{1}H} NMR (101 MHz, $CDCl_3$): δ = 142.9, 128.4, 128.2, 125.6, 35.7, 33.7, 22.4, 14.0 ppm.

RT (GC): 8.44 min MS: 134.17 m/z $[M]^+$ (Method **B**)

This data corresponds with previously reported data.[6]

N-Propylaniline (**3o**)

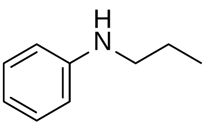

N-Allylaniline (120 µL, 0.88 mmol, 1 equiv.); catalyst (2.5 mg, 4.3 µmol, 0.5 mol%); KO^{t}Bu (5 mg, 43 µmol, 5 mol%); 118 mg (88%) of a colorless liquid

^{1}H NMR (400 MHz, $CDCl_3$): δ = 7.22 – 7.13 (m, 2H), 6.69 (tt, J = 7.3, 1.1 Hz, 1H), 6.65 – 6.56 (m, 2H), 3.09 (t, J = 7.2 Hz, 2H), 1.65 (h, J = 7.5 Hz, 2H), 1.00 (t, J = 7.4 Hz, 3H) ppm.

^{13}C{^{1}H} NMR (101 MHz, $CDCl_3$): δ = 148.7, 129.4, 117.2, 112.8, 45.9, 22.9, 11.8 ppm.

RT (GC): 4.09 min MS: 135.16 m/z $[M]^+$

This data corresponds with previously reported data.[7]

Ethylcyclohexane (**3p**)

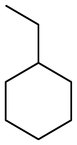

Vinylcyclohexane (121 µL, 0.88 mmol, 1 equiv.); catalyst (2.5 mg, 4.3 µmol, 0.5 mol%); KO^{t}Bu (5 mg, 43 µmol, 5 mol%); yield: 99 % (according to GC-MS with *n*-dodecane as standard)

RT (GC): 4.92 min MS: 112.19 m/z $[M]^+$ (Method **B**)

Ethylcyclohexane (**3q**)

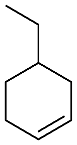

4-Vinylcyclohexene (116 µL, 0.88 mmol, 1 equiv.); catalyst (2.5 mg, 4.3 µmol, 0.5 mol%); KO^{t}Bu (5 mg, 43 µmol, 5 mol%); yield: 99 % (according to GC-MS with *n*-dodecane as standard)

RT (GC): 5.20 min MS: 110.17 m/z $[M]^+$ (Method **B**)

Dodecane (**3r**)

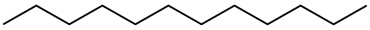

1-Dodecene (197 µL, 0.88 mmol, 1 equiv.); catalyst (1.25 mg, 2.2 µmol, 0.25 mol%); KO^{t}Bu (2.5 mg, 22 µmol, 2.5 mol%); yield: 88% (according to GC-MS with *n*-dodecane as standard) RT (GC): 3.91 min MS: 170.26 m/z $[M]^+$

Chlorohexane (**3s**)

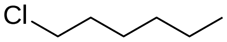

6-Chloro-1-hexene (117 µL, 0.88 mmol, 1 equiv.); catalyst (1.25 mg, 2.2 µmol, 0.25 mol%); KO^{t}Bu (2.5 mg, 22 µmol, 2.5 mol%); yield: 91 % (according to GC-MS with *n*-dodecane as standard)

RT (GC): 5.14 min MS: 120.14 m/z $[M]^+$ (Method **B**)

Dipropylamine (**3t**)

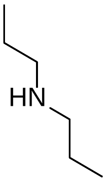

Diallylamine (109 µL, 0.88 mmol, 1 equiv.); catalyst (2.5 mg, 4.3 µmol, 0.5 mol%); KO^{t}Bu (5 mg, 43 µmol, 5 mol%); yield: 99% (according to NMR with dibromomethane as standard)

Propylbenzene (**3u**)

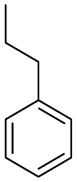

Allylbenzene (118 µL, 0.88 mmol, 1 equiv.); catalyst (2.5 mg, 4.3 µmol, 0.5 mol%); KO^{t}Bu (5 mg, 43 µmol, 5 mol%); yield: 40% (according to GC-MS with *n*-dodecane as standard)

RT (GC): 6.89 min MS: 120.17 m/z $[M]^+$ (Method **B**)

2-Propylanisole (**3v**)

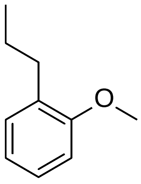

2-Allylanisole (137 µL, 0.88 mmol, 1 equiv.); catalyst (2.5 mg, 4.3 µmol, 0.5 mol%); KO^{t}Bu (5 mg, 43 µmol, 5 mol%); yield: 54 % (according to GC-MS with *n*-dodecane as standard)

RT (GC): 3.72 min MS: 150.17 m/z $[M]^+$

6 References

(1) Eizawa, A.; Arashiba, K.; Tanaka, H.; Kuriyama, S.; Matsuo, Y.; Nakajima, K.; Yoshizawa, K.; Nishibayashi, Y. *Nat. Commun.* **2017**, *8*, 14874.

(2) Arashiba, K.; Itabashi, T.; Nakajima, K.; Nishibayashi, Y. *Chem. Lett.* **2019**, *48*, 693–695.

(3) Griffin, J. D.; Zeller, M. A.; Nicewicz, D. A.. *J. Am. Chem. Soc.* **2015**, *137*, 11340–11348.

(4) Espinal-Viguri, M.; Neale, S. E.; Coles, N. T.; Macgregor, S. A.; Webster, R. L. *J. Am. Chem. Soc.* **2019**, *141*, 572–582.

(5) Zhao Ming; Huang, Binbin; Yang, Chao; Gao, Yuan; Xia, Wujiong, X. C. *Synthesis (Stuttg).* **2018**, *50*, 2981–2989.

(6) Mastalir, M.; Pittenauer, E.; Stöger, B.; Allmaier, G.; Kirchner, K. *Org. Lett.* **2017**, *19*, 2178–2181.

(7) Zou, Y.-Q.; Chakraborty, S.; Nerush, A.; Oren, D.; Diskin-Posner, Y.; Ben-David, Y.; Milstein, D. *ACS Catal.* **2018**, *8*, 8014–8019.

7 NMR and IR Spectra

7.1 Ligand Spectra

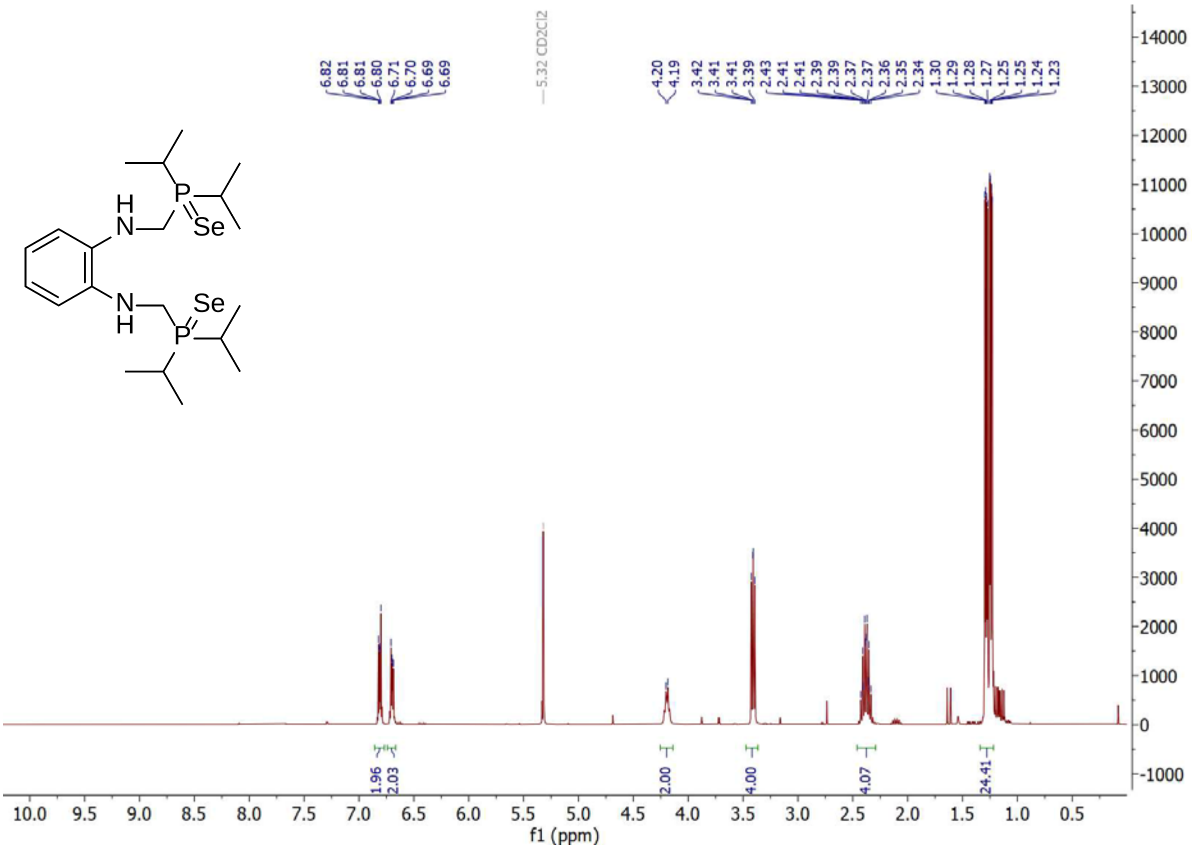

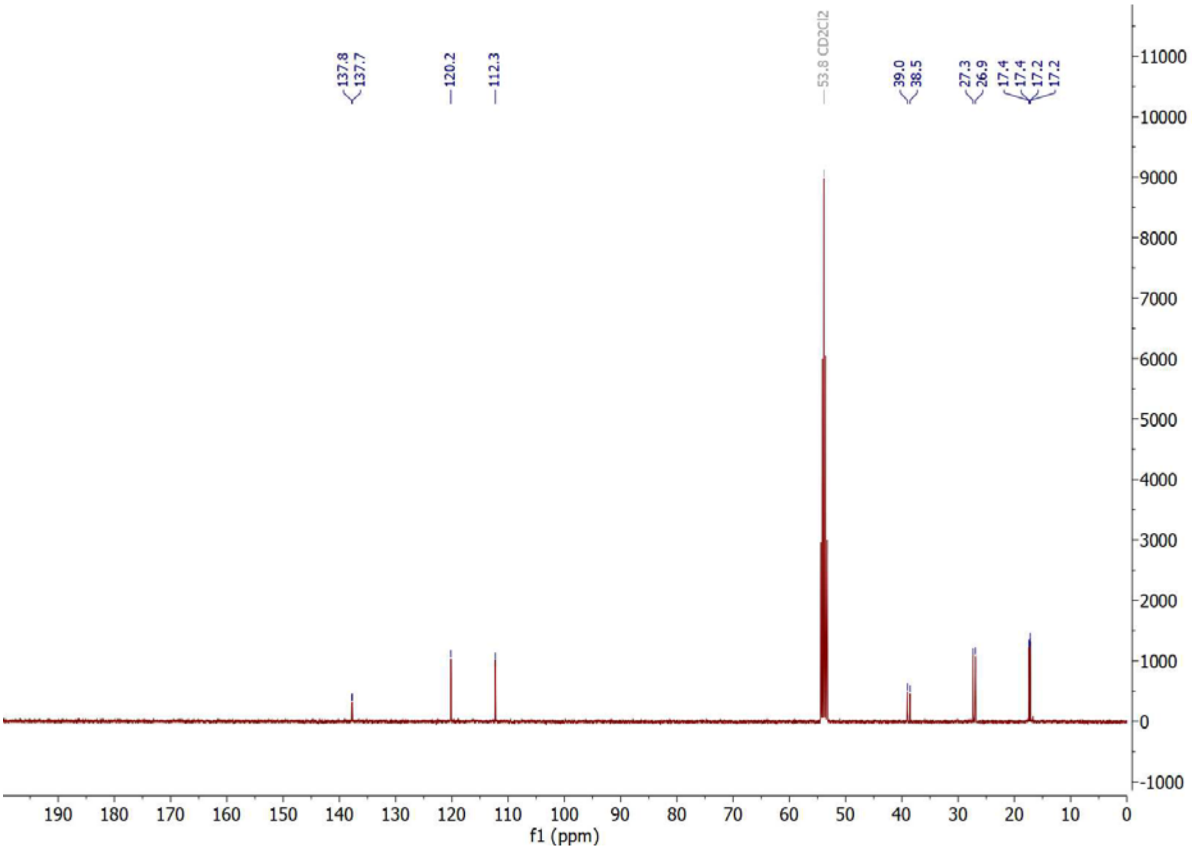

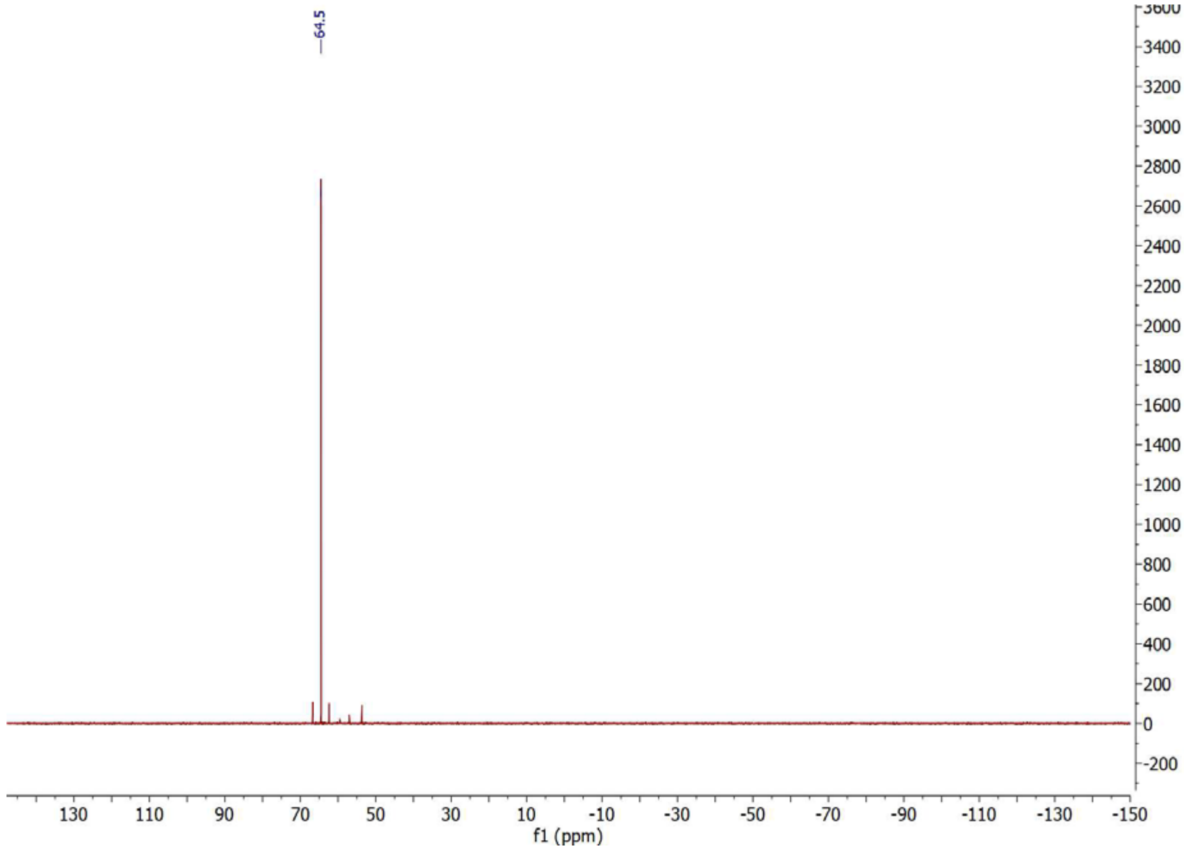
64.5
f1 (ppm)

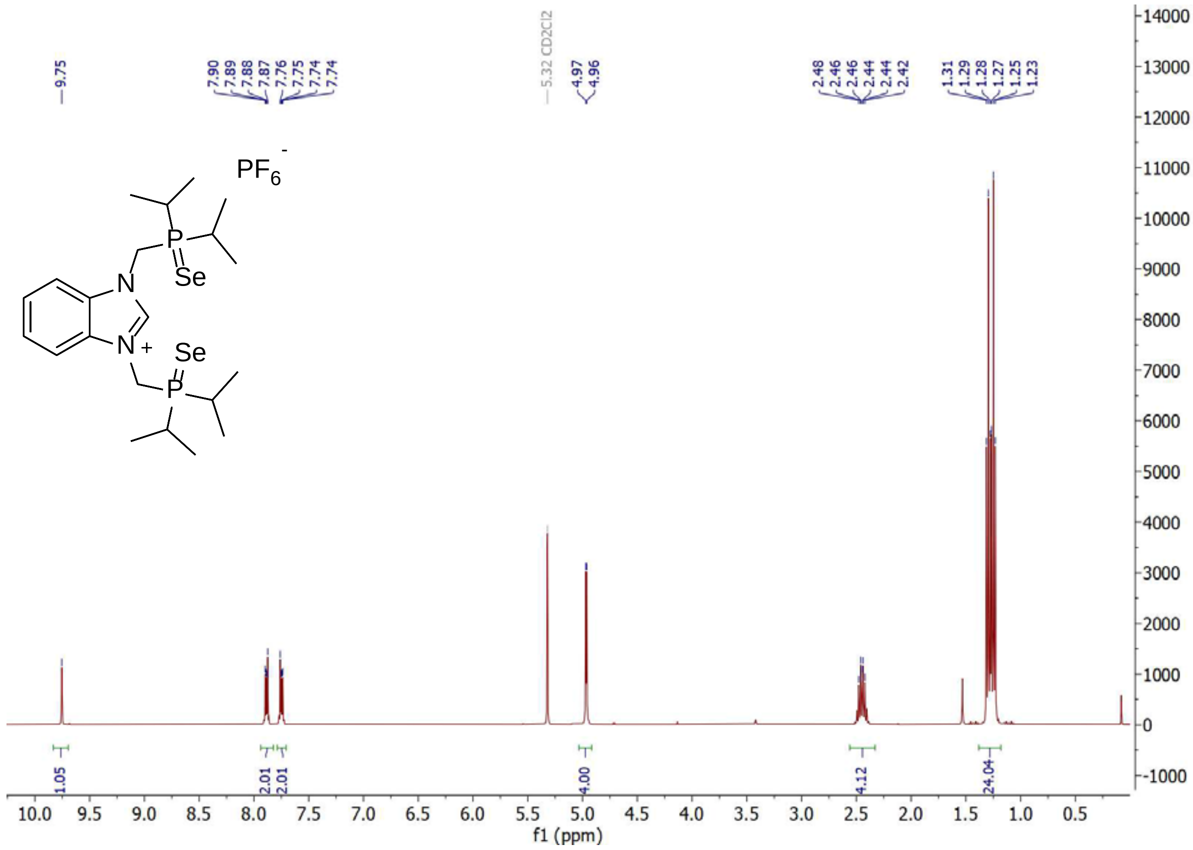
9.75
7.90 7.89 7.88 7.87 7.76 7.75 7.74 7.74
5.32 CD2Cl2
4.97 4.96
2.48 2.46 2.46 2.44 2.44 2.42
1.31 1.29 1.28 1.27 1.25 1.23
PF6-
P
Se
N
N+
Se
P
1.05
2.01
2.01
4.00
4.12
24.04
f1 (ppm)

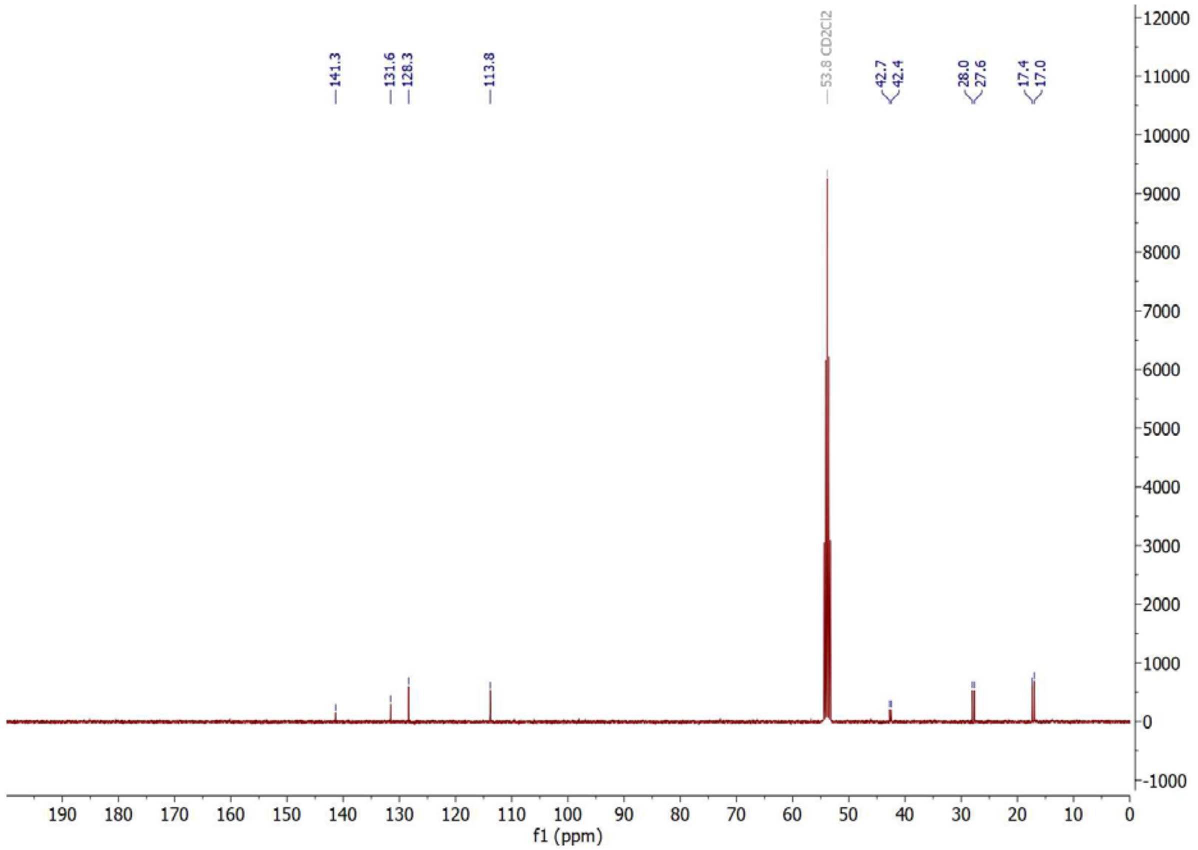
141.3
131.6
128.3
113.8
53.8 CD2Cl2
42.7
42.4
28.0
27.6
17.4
17.0
f1 (ppm)

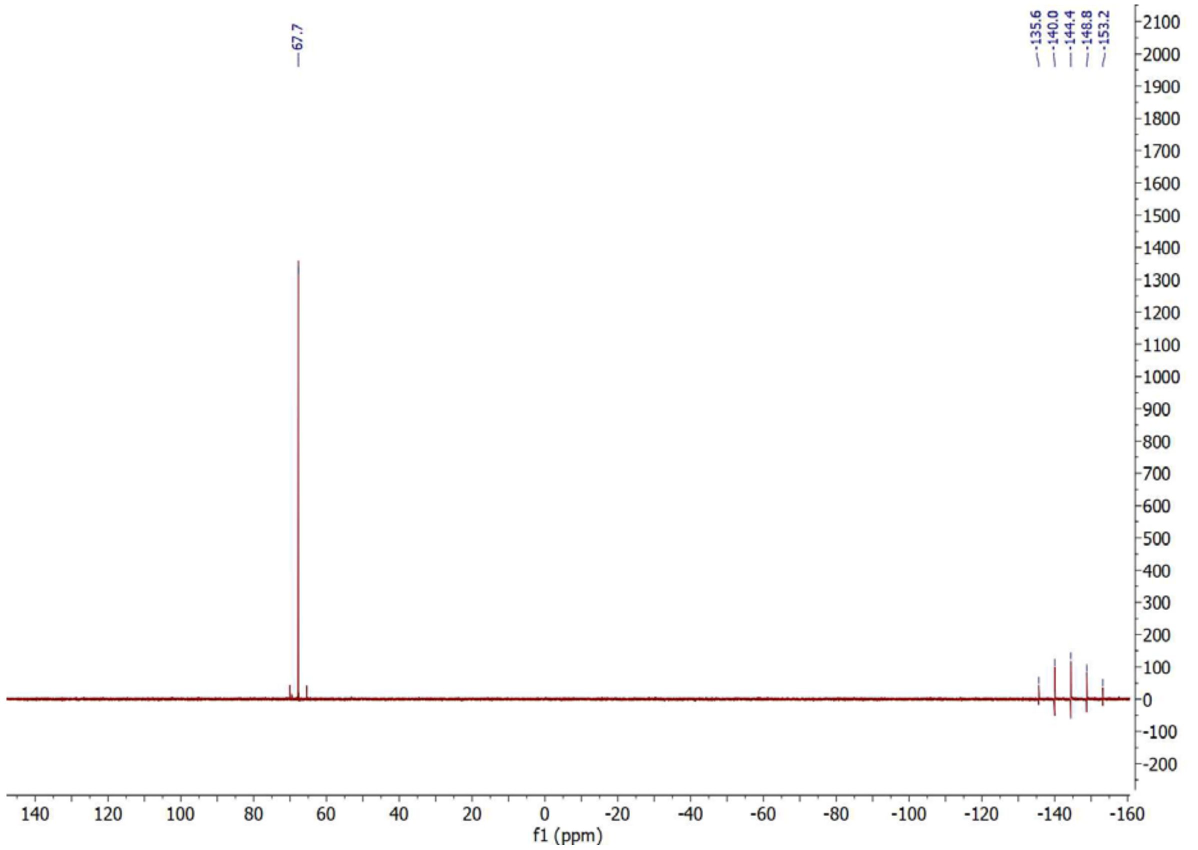
67.7
-135.6
-140.0
-144.4
-148.8
-153.2
f1 (ppm)

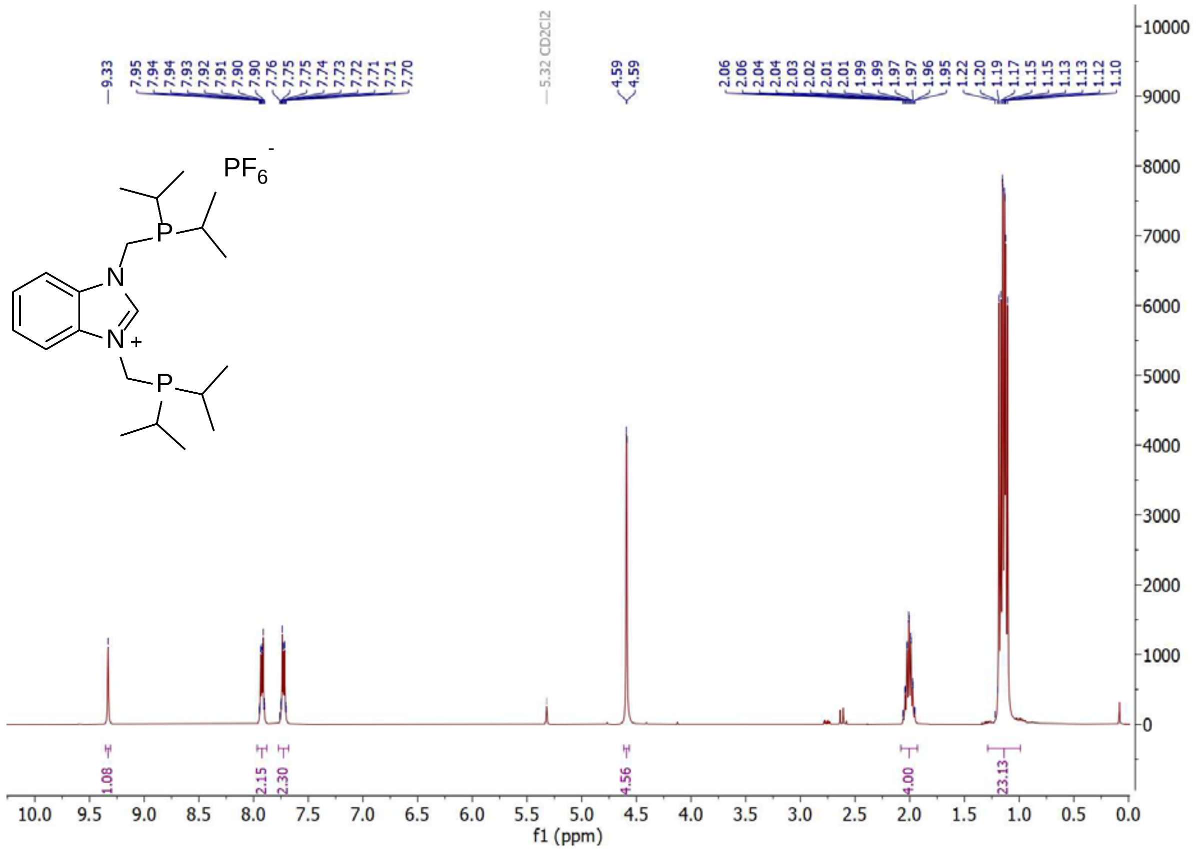
9.33
7.95
7.94
7.94
7.93
7.92
7.91
7.90
7.90
7.76
7.75
7.75
7.74
7.73
7.72
7.71
7.71
7.70
5.32 CD2Cl2
4.59
4.59
2.06
2.06
2.04
2.04
2.03
2.02
2.01
2.01
1.99
1.99
1.97
1.97
1.96
1.95
1.22
1.20
1.19
1.17
1.15
1.15
1.13
1.13
1.12
1.10
PF6 -
P
N
N+
P
1.08
2.15
2.30
4.56
4.00
23.13
f1 (ppm)

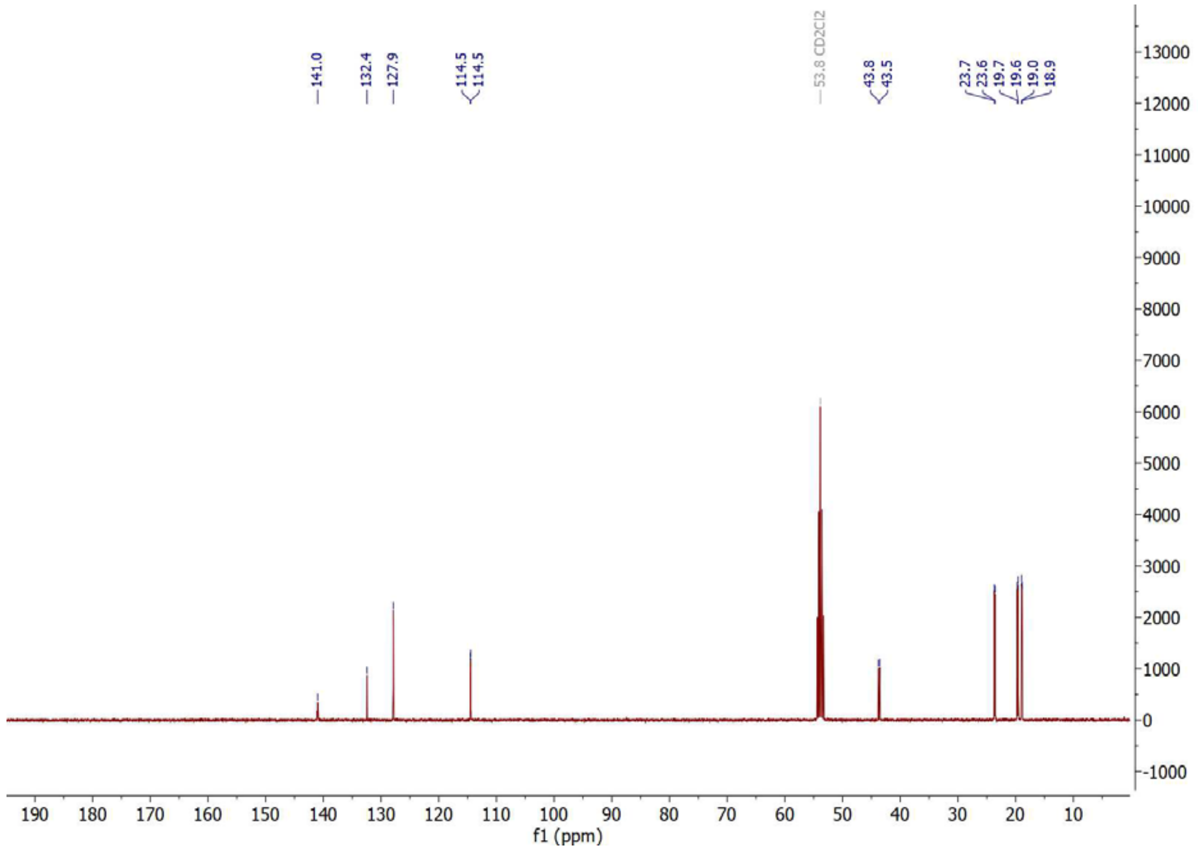
141.0
132.4
127.9
114.5
114.5
53.8 CD2Cl2
43.8
43.5
23.7
23.6
19.7
19.6
19.0
18.9
f1 (ppm)

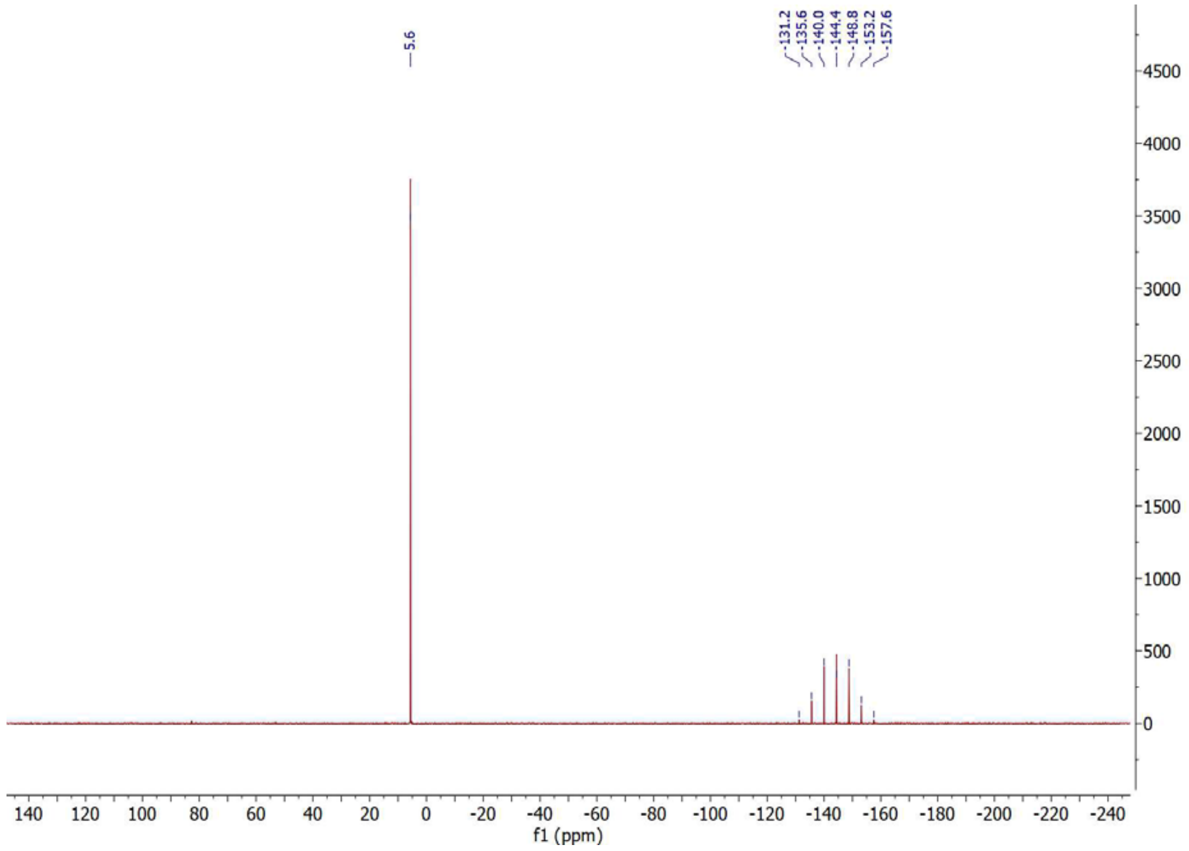

7.2 Complex Spectra

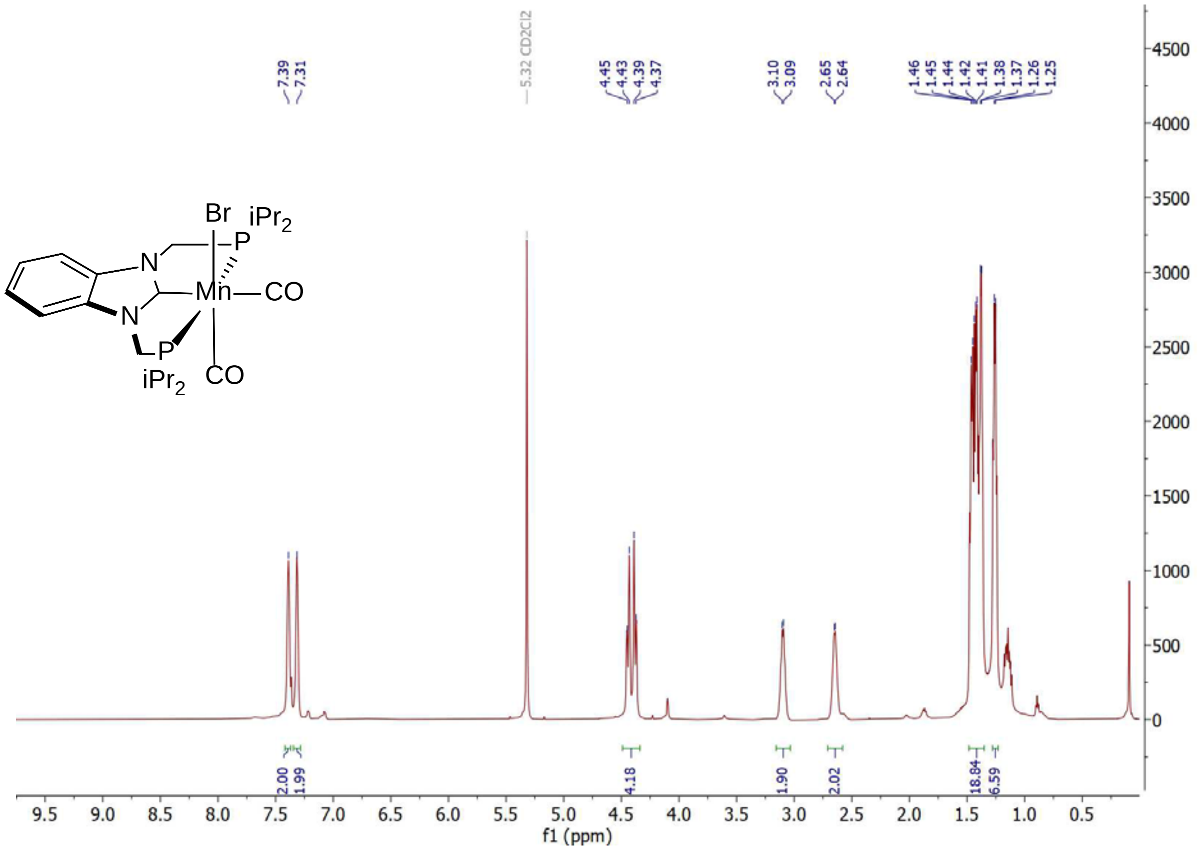

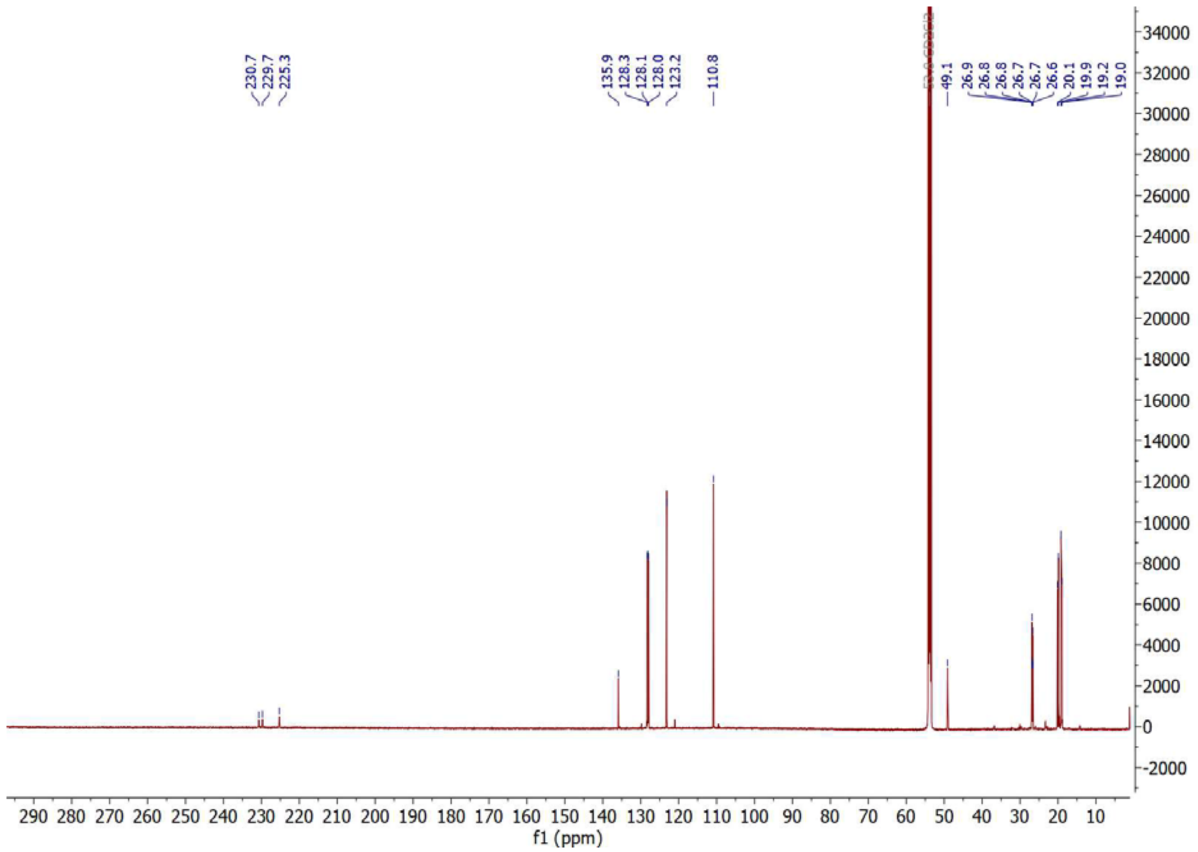
230.7
229.7
225.3
135.9
128.3
128.1
128.0
123.2
110.8
49.1
26.9
26.8
26.8
26.7
26.7
26.6
20.1
19.9
19.2
19.0
f1 (ppm)

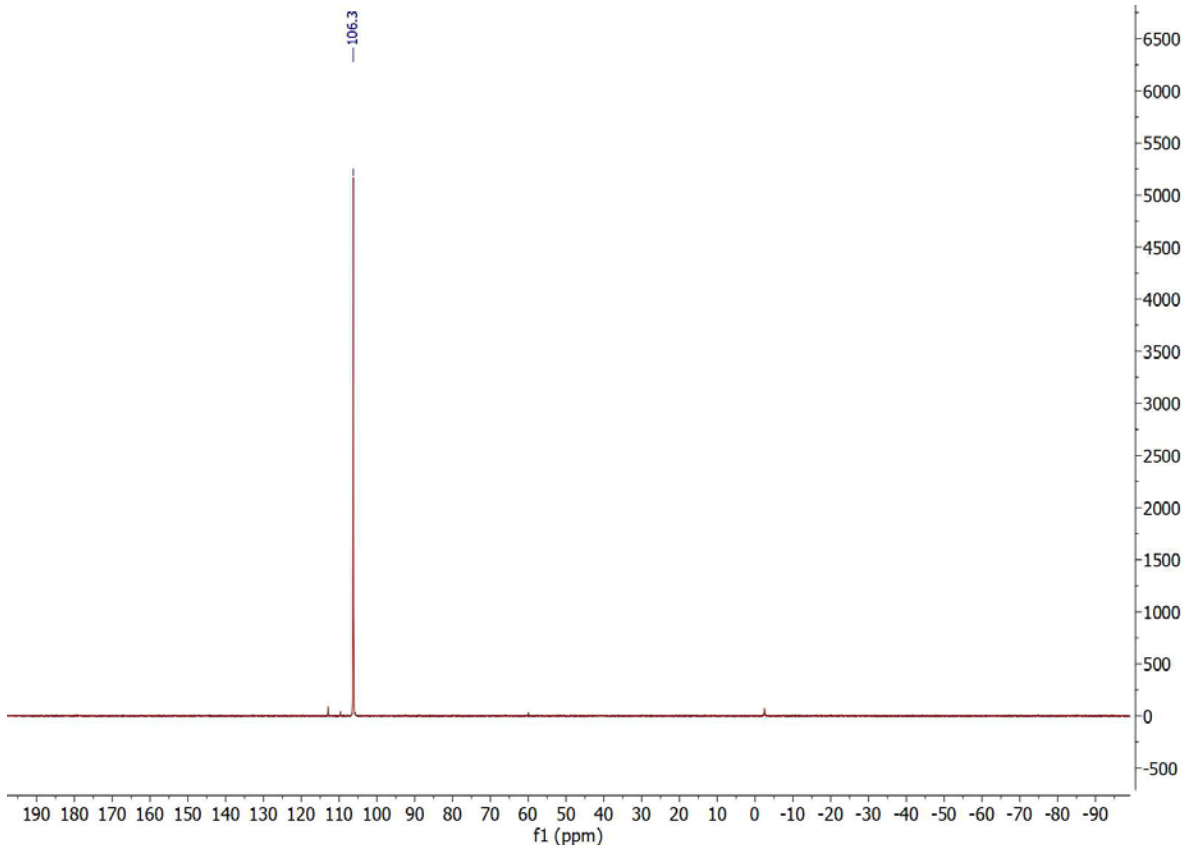
106.3
f1 (ppm)

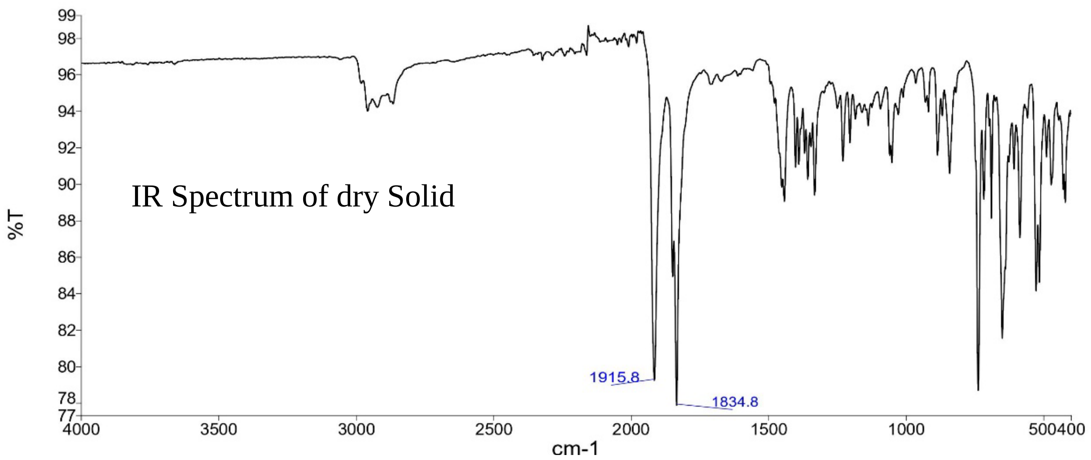
IR Spectrum of dry Solid
%T
99
98
96
94
92
90
88
86
84
82
80
78
77
4000
3500
3000
2500
2000
1500
1000
500400
cm-1
1915.8
1834.8

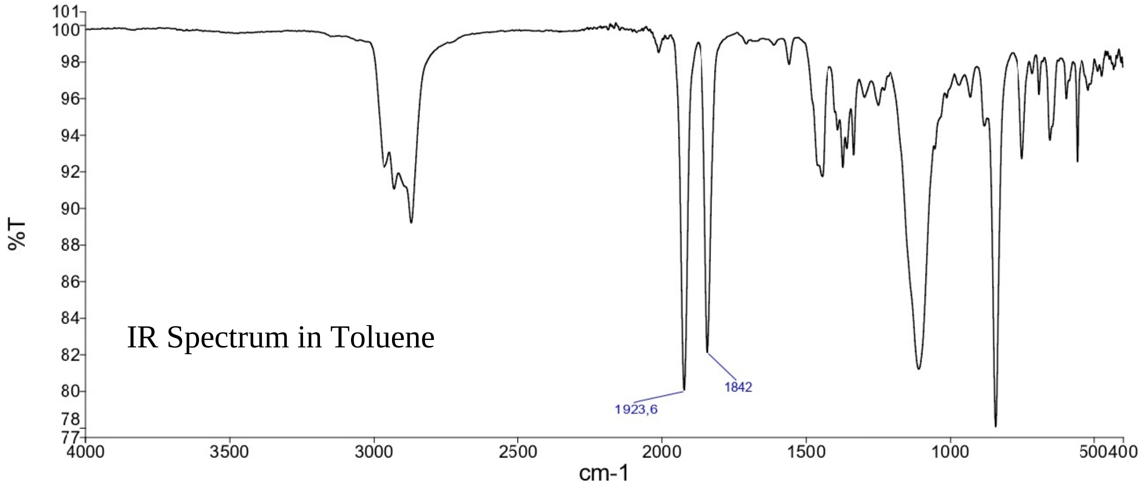
IR Spectrum in Toluene
%T
101
100
98
96
94
92
90
88
86
84
82
80
78
77
4000
3500
3000
2500
2000
1500
1000
500400
cm-1
1923,6
1842

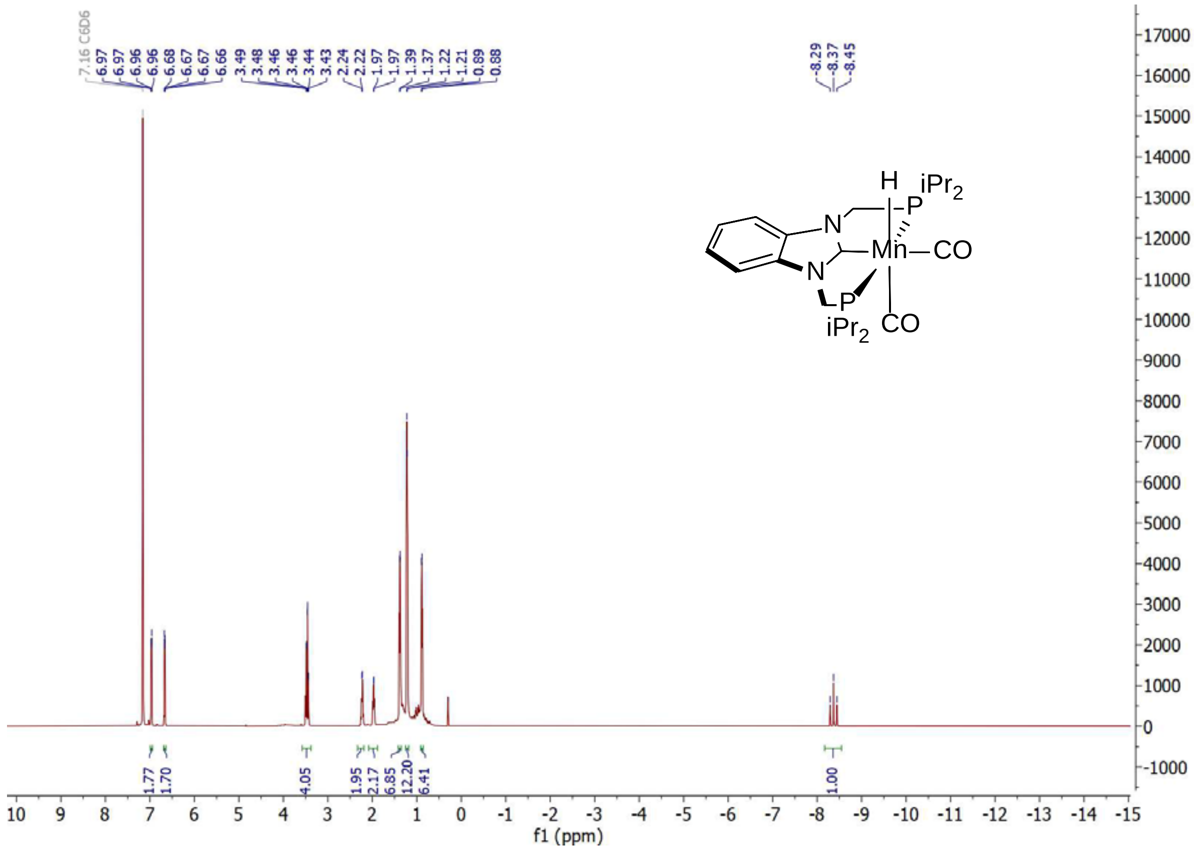
7.16 C6D6
6.97
6.97
6.96
6.96
6.68
6.67
6.67
6.66
3.49
3.48
3.46
3.46
3.44
3.43
2.24
2.22
1.97
1.97
1.39
1.37
1.22
1.21
0.89
0.88
-8.29
-8.37
-8.45
1.77
1.70
4.05
1.95
2.17
6.85
12.20
6.41
1.00
f1 (ppm)
H
iPr2
P
N
N
Mn
CO
CO
P
iPr2

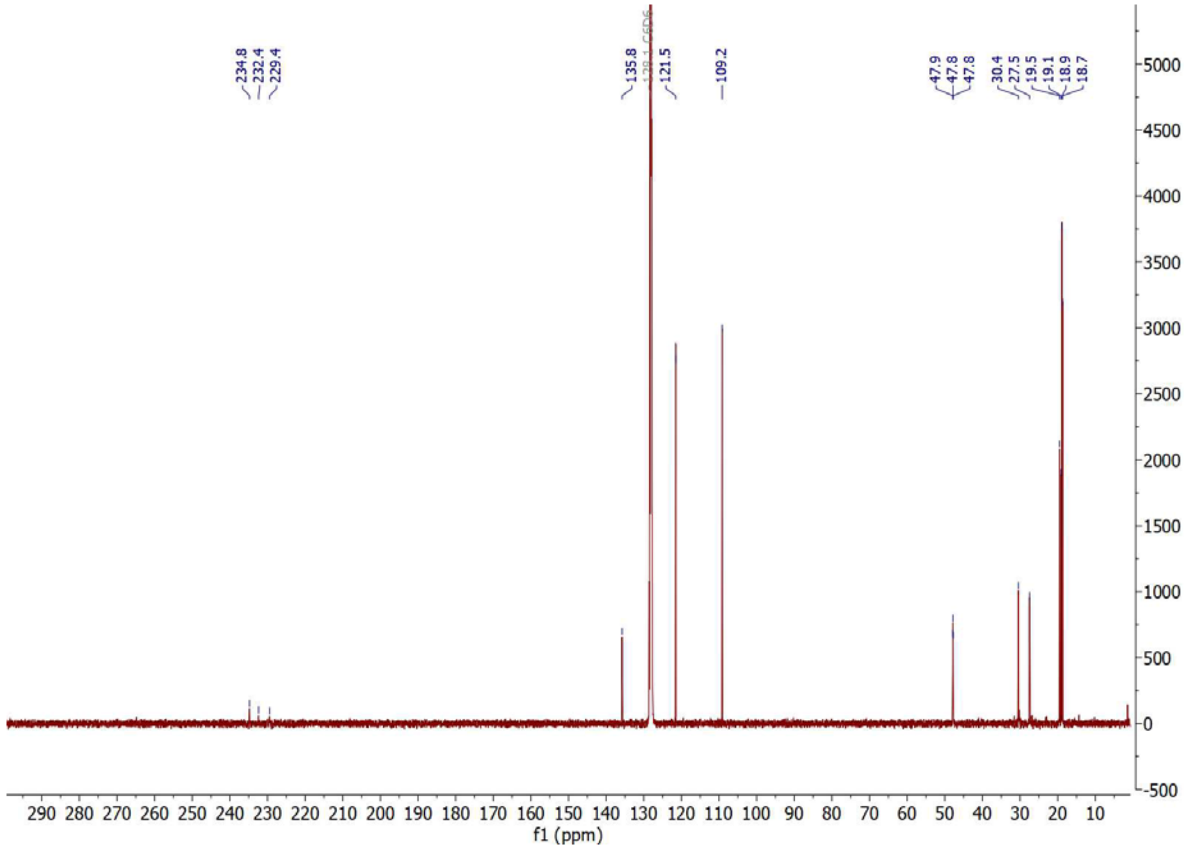
234.8
232.4
229.4
135.8
128.1 C6D6
121.5
109.2
47.9
47.8
47.8
30.4
27.5
19.5
19.1
18.9
18.7
f1 (ppm)

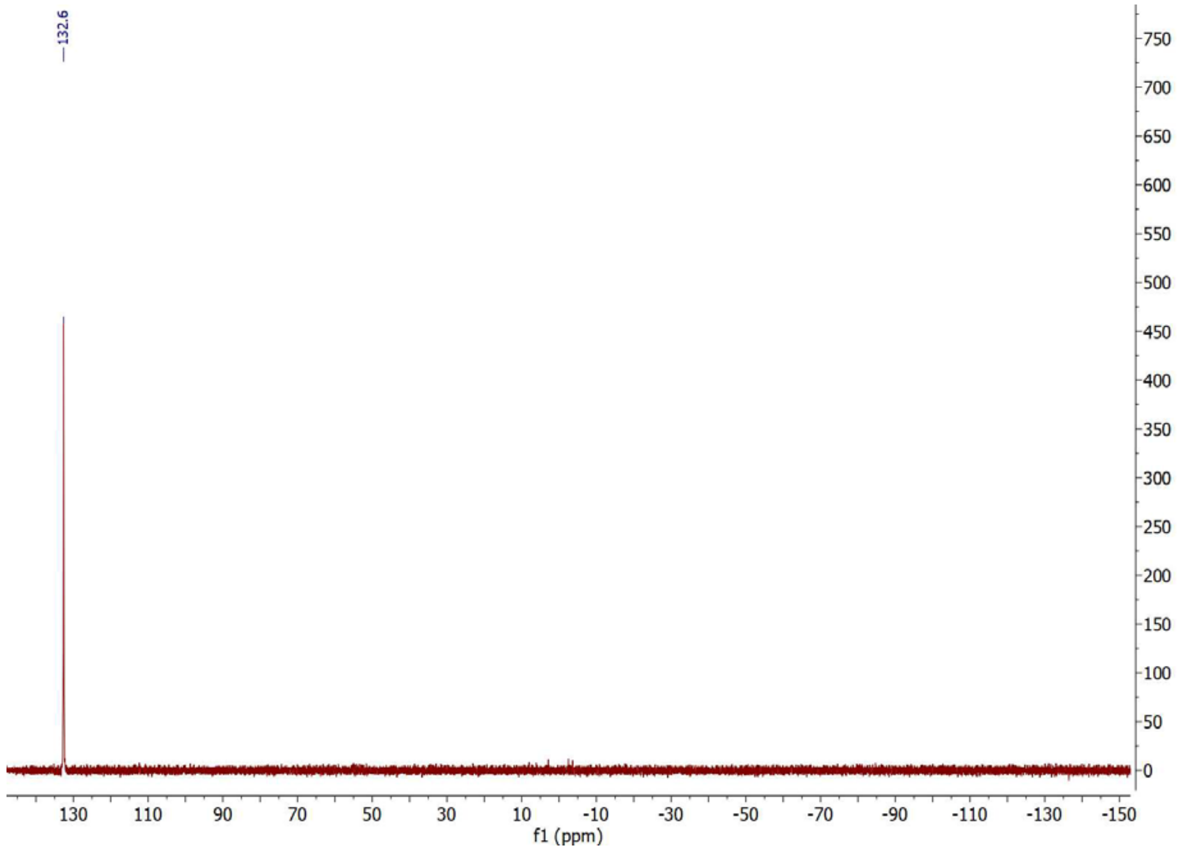
132.6
f1 (ppm)

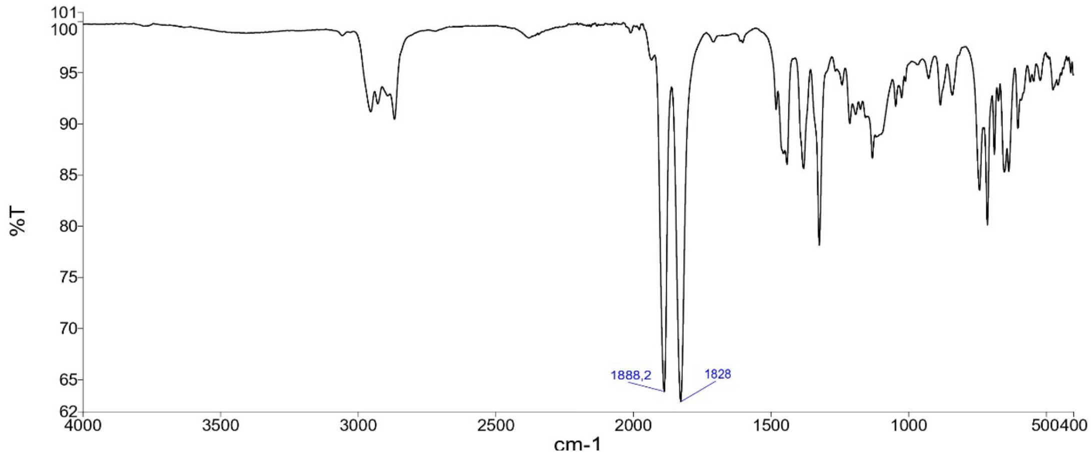
%T
cm-1
1888,2
1828

7.3 Spectra of Organic Products

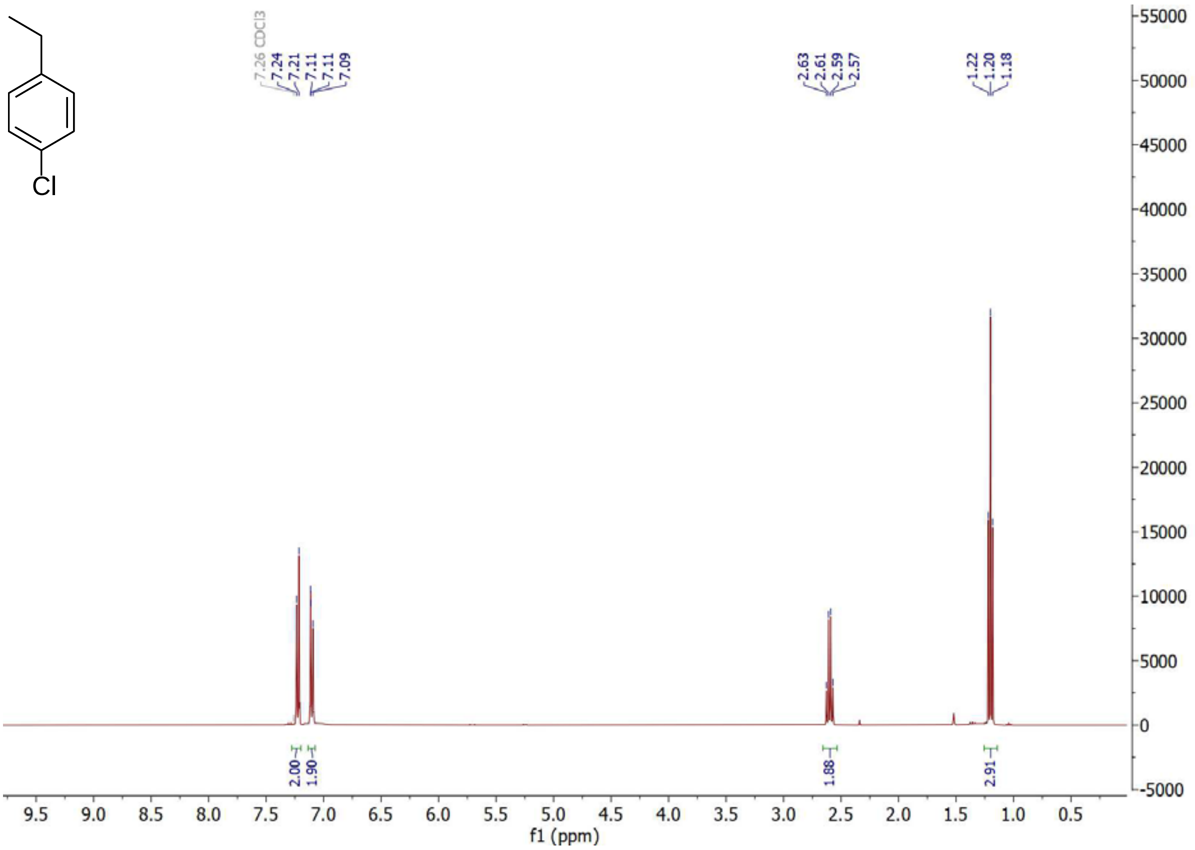

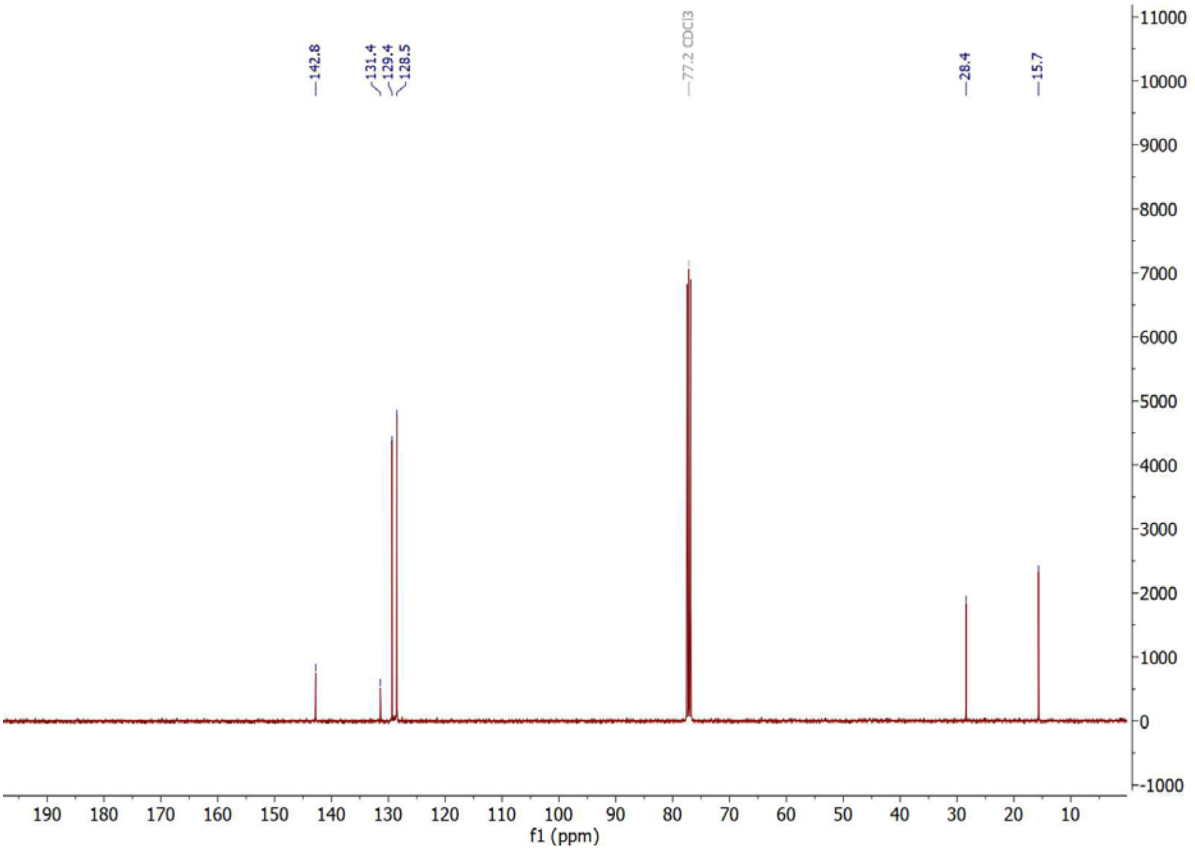

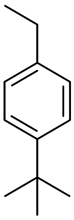

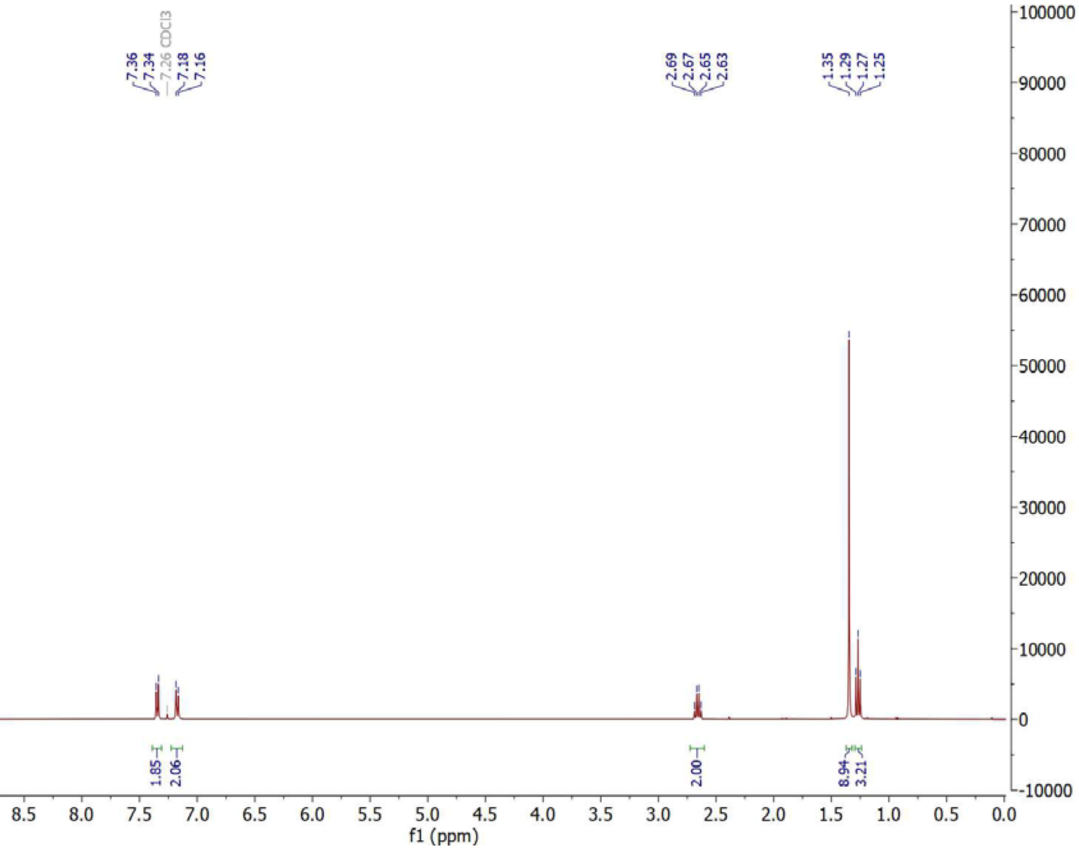
7.36
7.34
7.26 CDCl3
7.18
7.16
2.69
2.67
2.65
2.63
1.35
1.29
1.27
1.25
1.85
2.06
2.00
8.94
3.21
f1 (ppm)

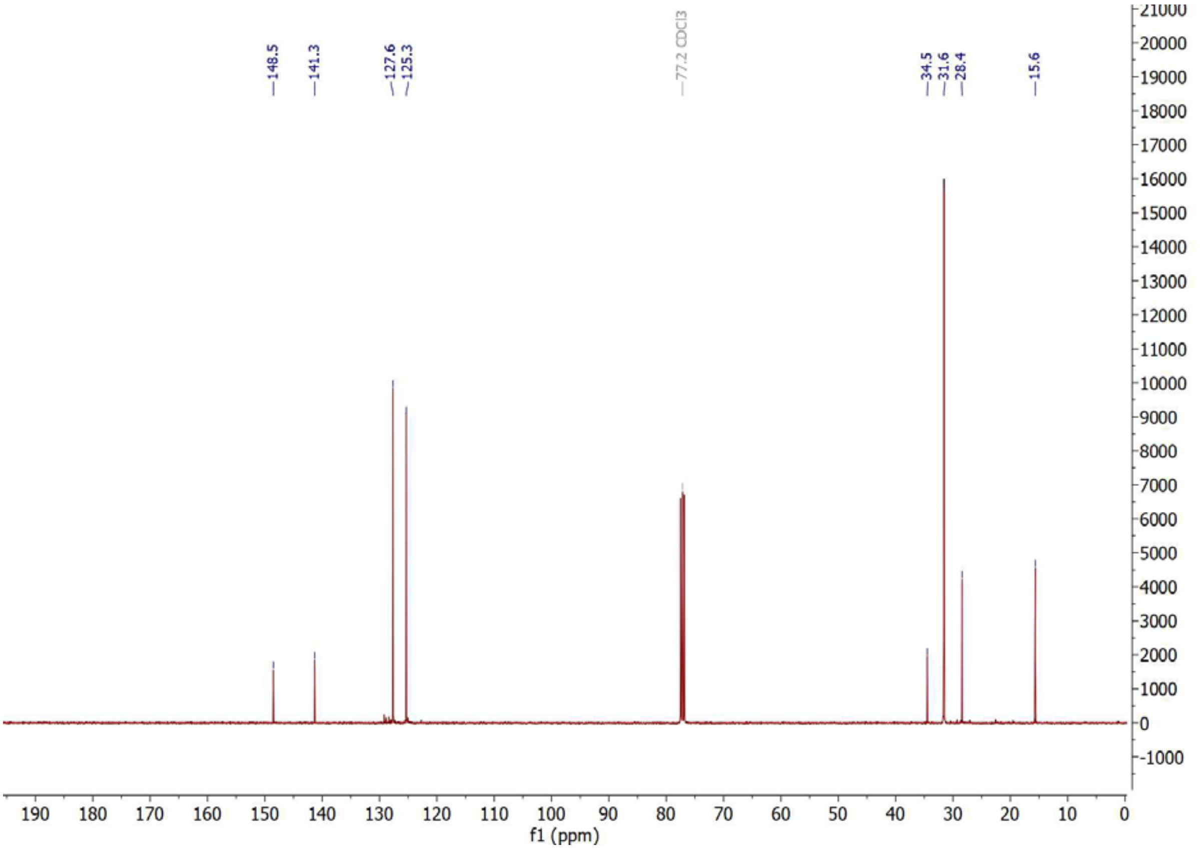
148.5
141.3
127.6
125.3
77.2 CDCl3
34.5
31.6
28.4
15.6
f1 (ppm)

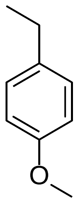
O

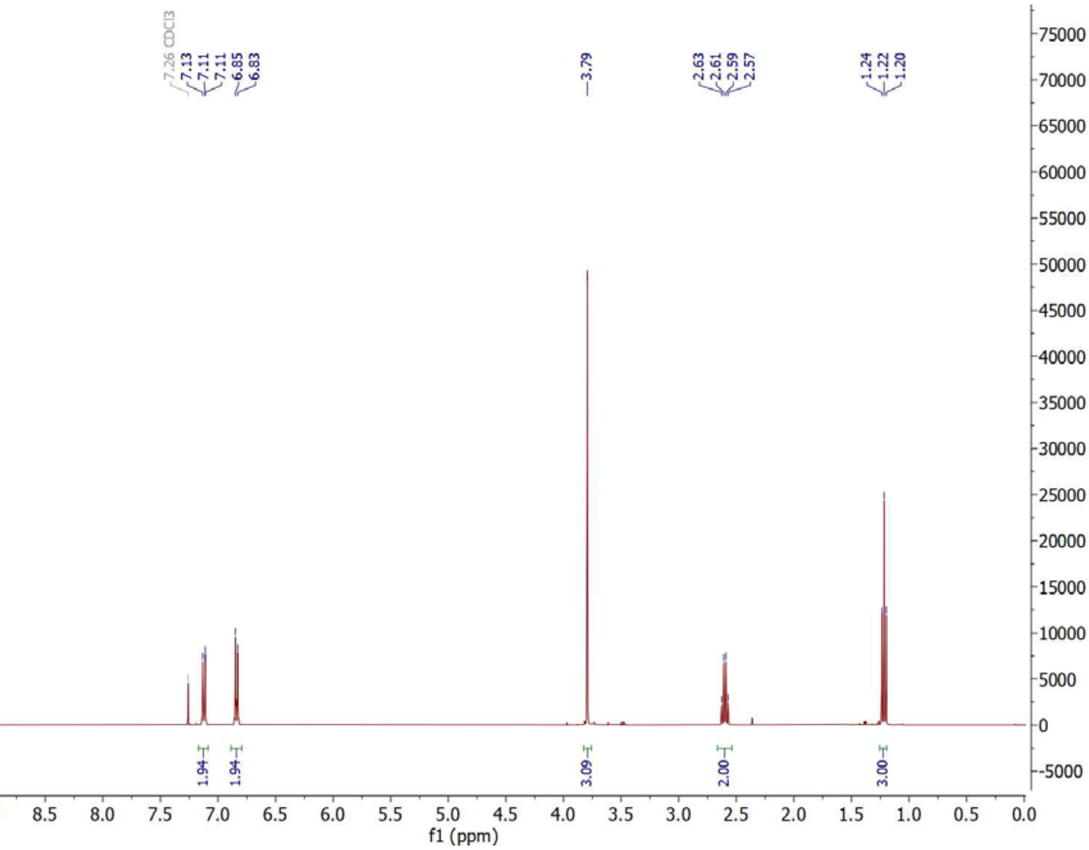
7.26 CDCl3
7.13
7.11
7.11
6.85
6.83
3.79
2.63
2.61
2.59
2.57
1.24
1.22
1.20
1.94
1.94
3.09
2.00
3.00
f1 (ppm)

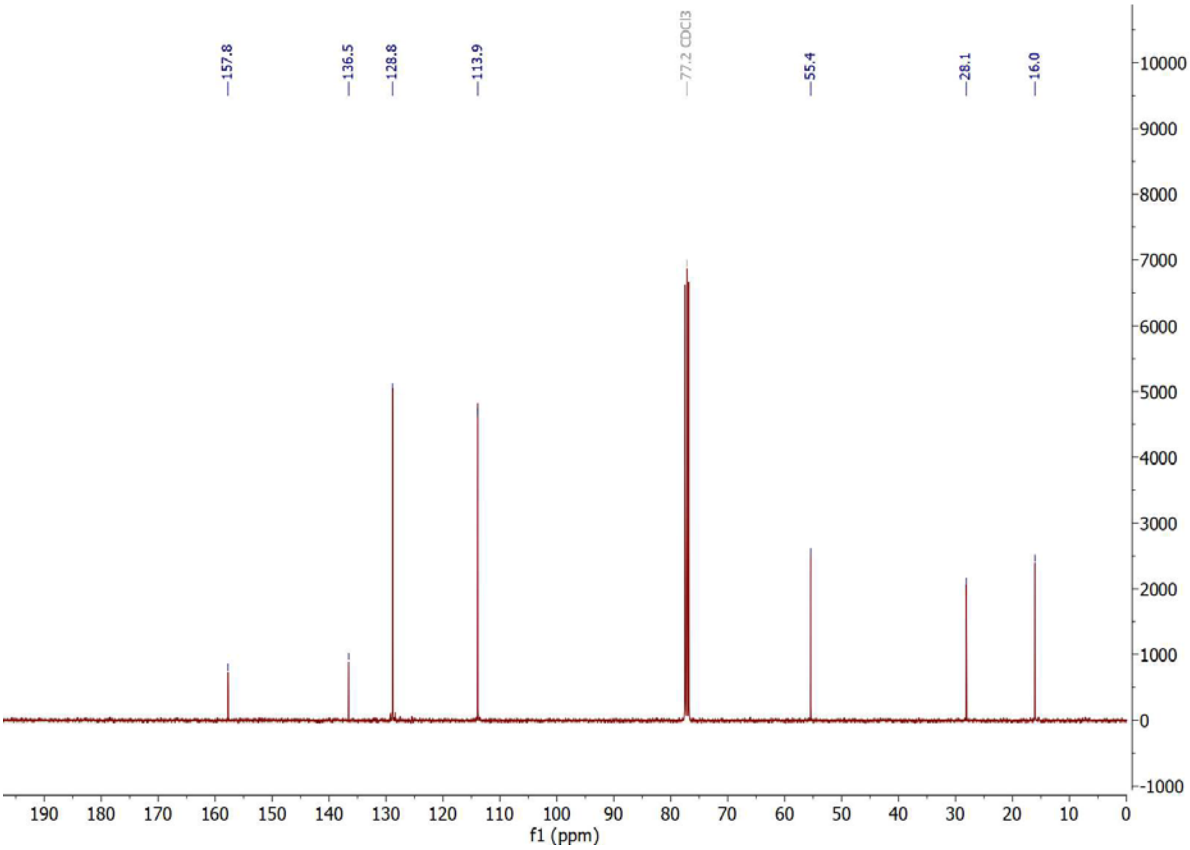
157.8
136.5
128.8
113.9
77.2 CDCl3
55.4
28.1
16.0
f1 (ppm)

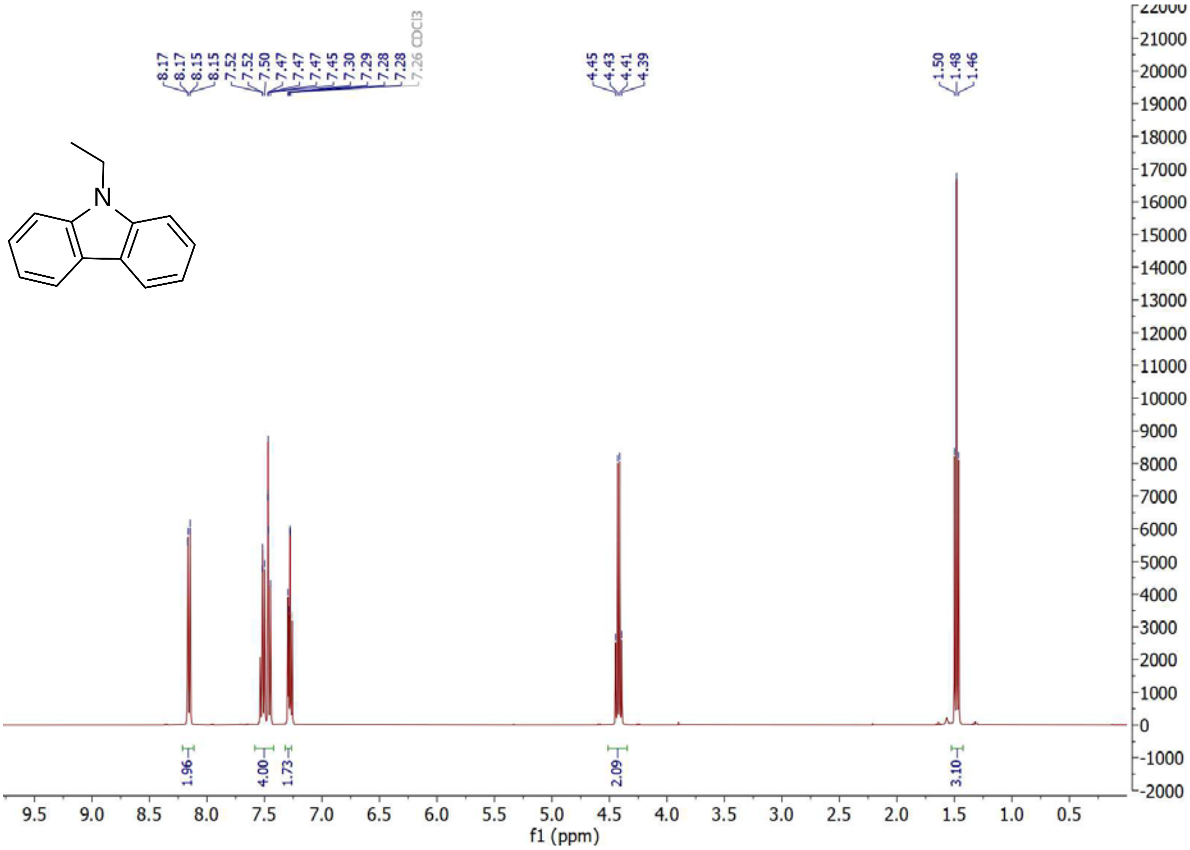
N
8.17
8.17
8.15
8.15
7.52
7.52
7.50
7.47
7.47
7.47
7.45
7.30
7.29
7.28
7.28
7.26 CDCl3
4.45
4.43
4.41
4.39
1.50
1.48
1.46
1.96
4.00
1.73
2.09
3.10
f1 (ppm)

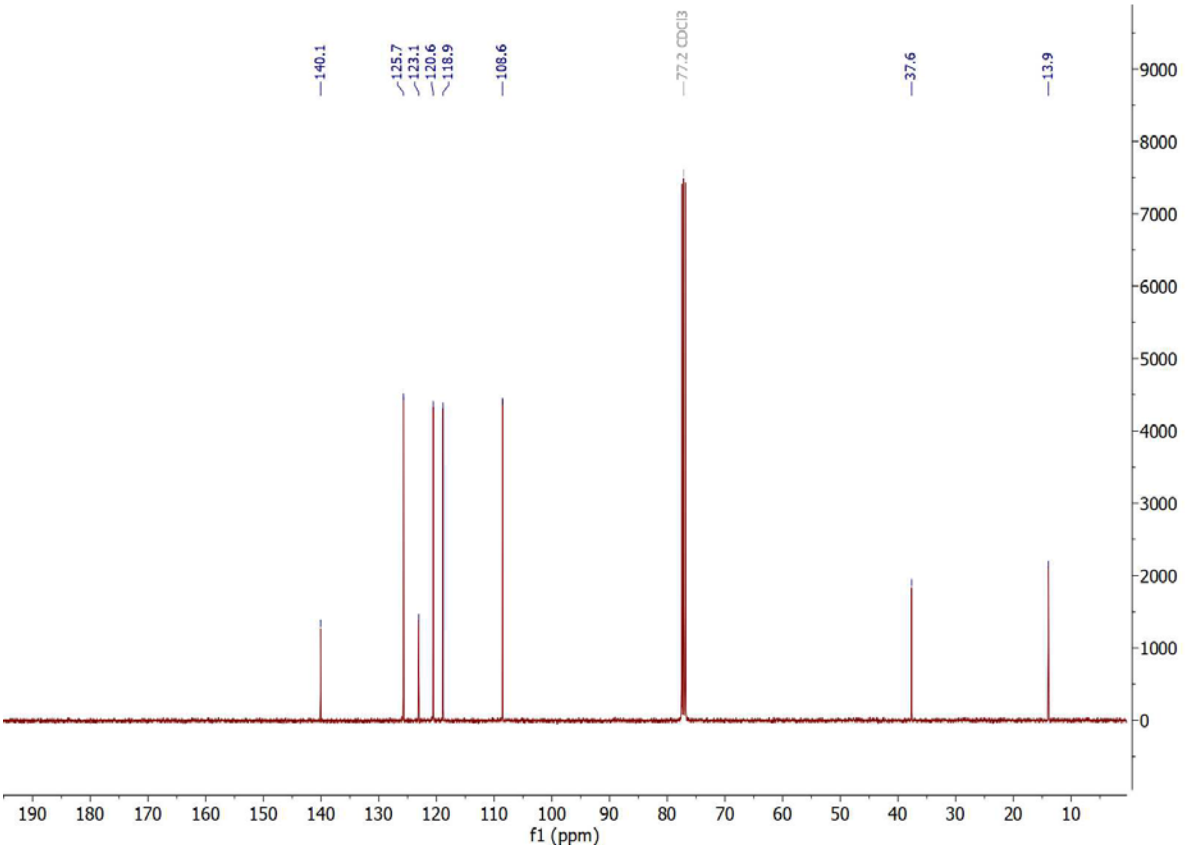
140.1
125.7
123.1
120.6
118.9
108.6
77.2 CDCl3
37.6
13.9
f1 (ppm)

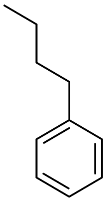

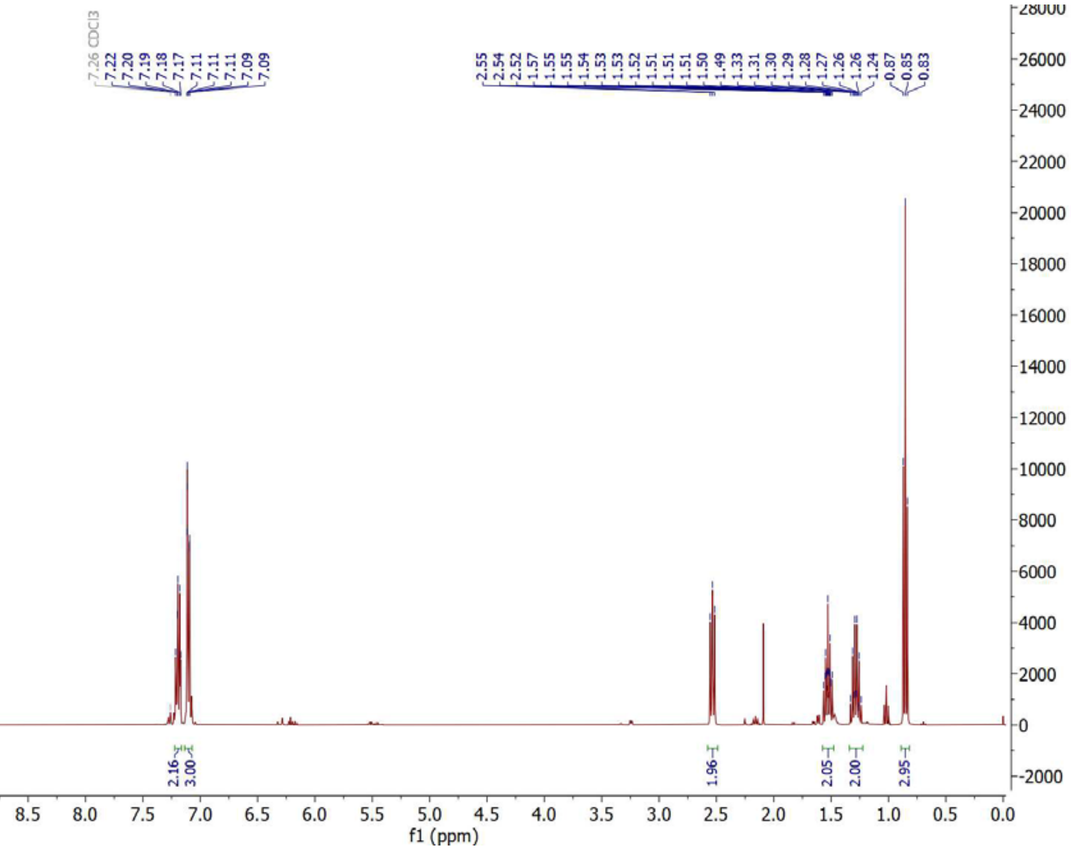
7.26 CDCl3
7.22
7.20
7.19
7.18
7.17
7.11
7.11
7.11
7.09
7.09
2.55
2.54
2.52
1.57
1.55
1.55
1.54
1.53
1.53
1.52
1.51
1.51
1.51
1.50
1.49
1.33
1.31
1.30
1.29
1.28
1.27
1.26
1.26
1.24
0.87
0.85
0.83
2.16
3.00
1.96
2.05
2.00
2.95
f1 (ppm)

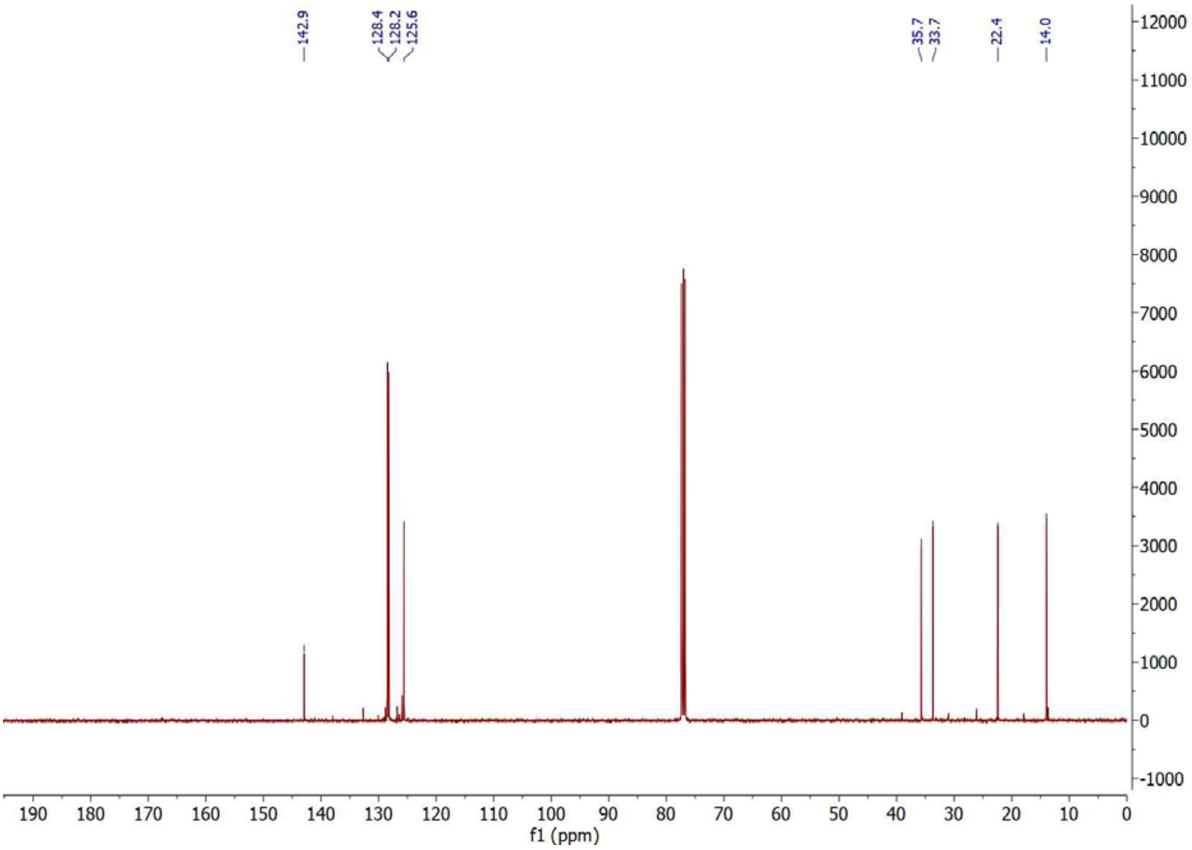
142.9
128.4
128.2
125.6
35.7
33.7
22.4
14.0
f1 (ppm)

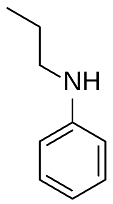

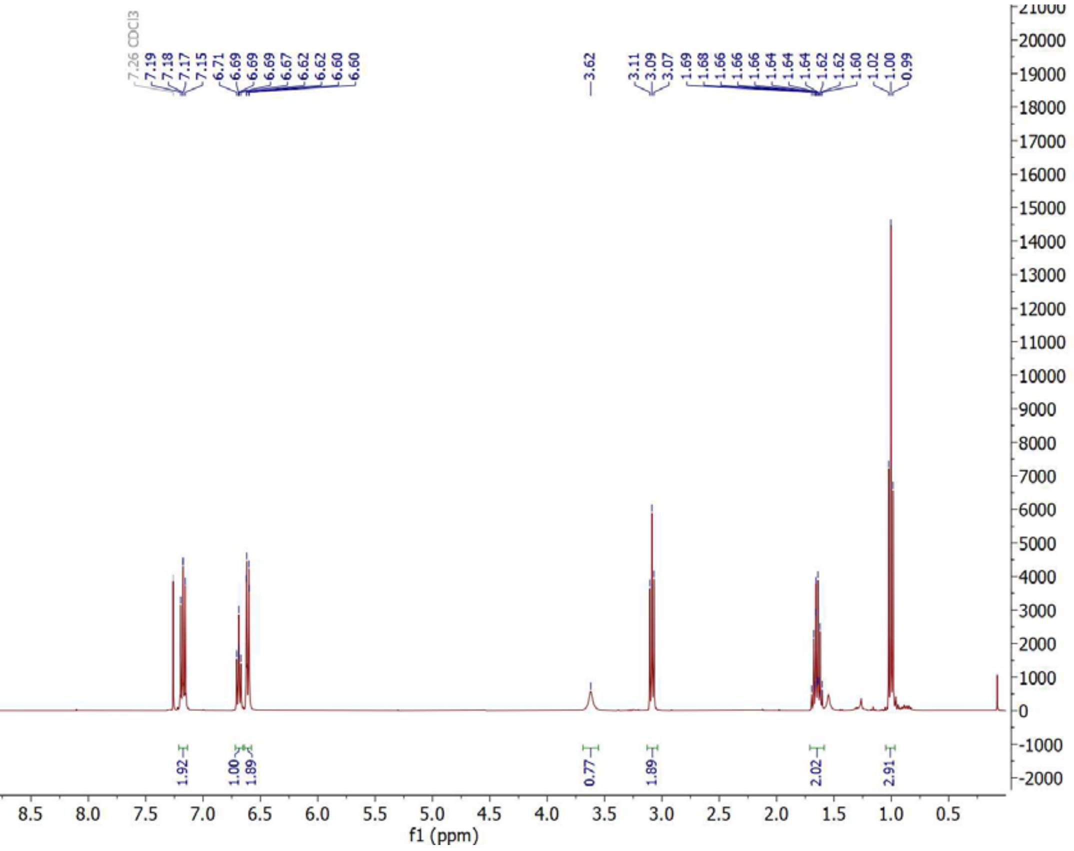
7.26 CDCl3
7.19
7.18
7.17
7.15
6.71
6.69
6.69
6.67
6.62
6.62
6.60
6.60
3.62
3.11
3.09
3.07
1.69
1.68
1.66
1.66
1.64
1.64
1.64
1.62
1.62
1.60
1.02
1.00
0.99
1.92
1.00
1.89
0.77
1.89
2.02
2.91
f1 (ppm)

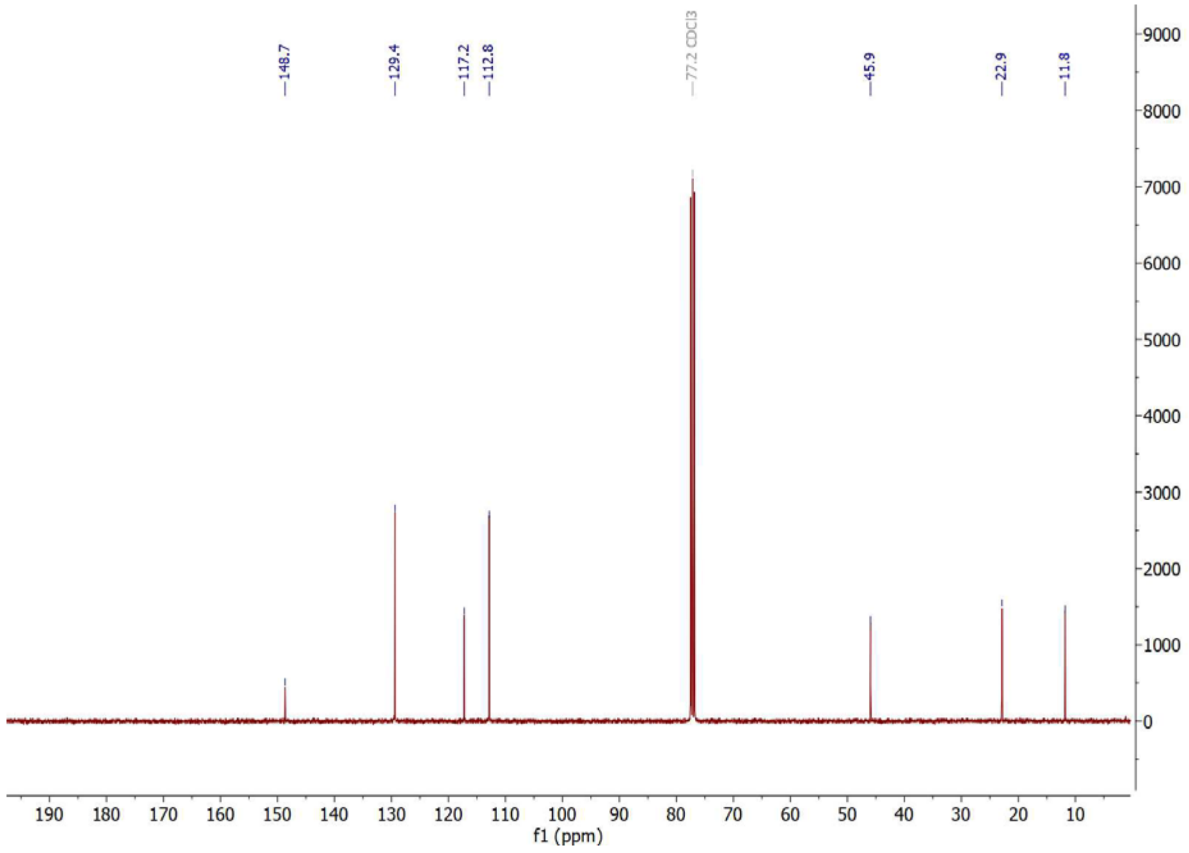
148.7
129.4
117.2
112.8
77.2 CDCl3
45.9
22.9
11.8
f1 (ppm)

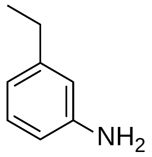
NH2

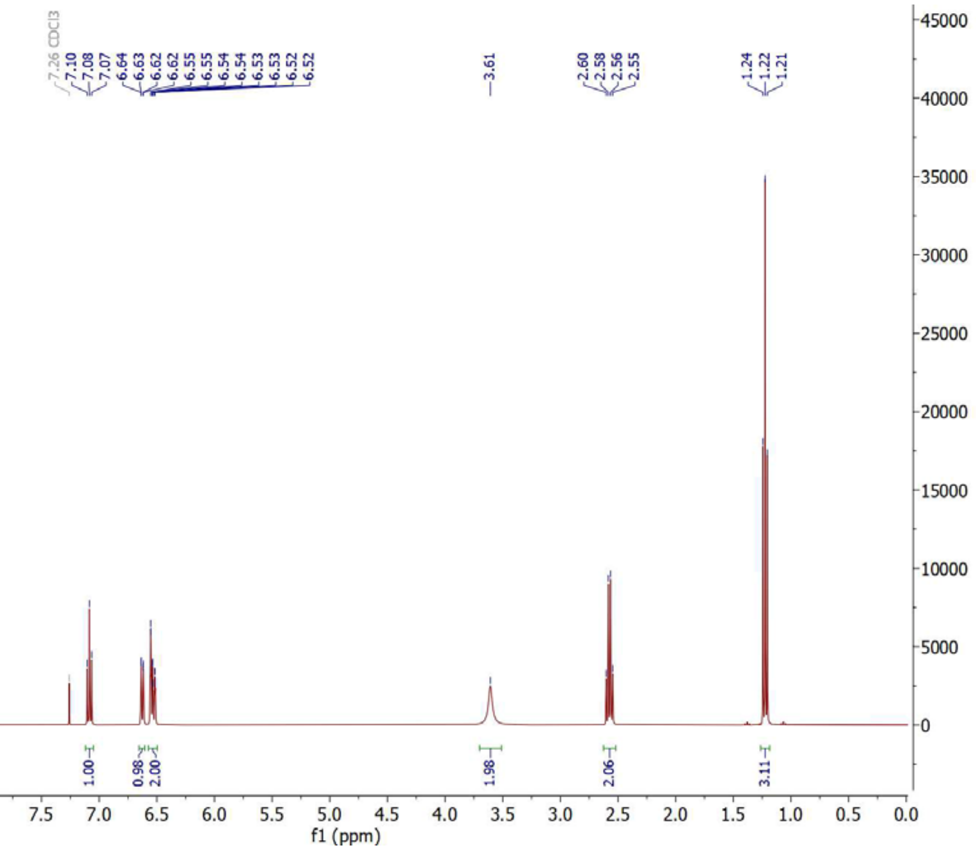
7.26 CDCl3
7.10
7.08
7.07
6.64
6.63
6.62
6.62
6.55
6.55
6.54
6.54
6.53
6.53
6.52
6.52
3.61
2.60
2.58
2.56
2.55
1.24
1.22
1.21
1.00
0.98
2.00
1.98
2.06
3.11
f1 (ppm)

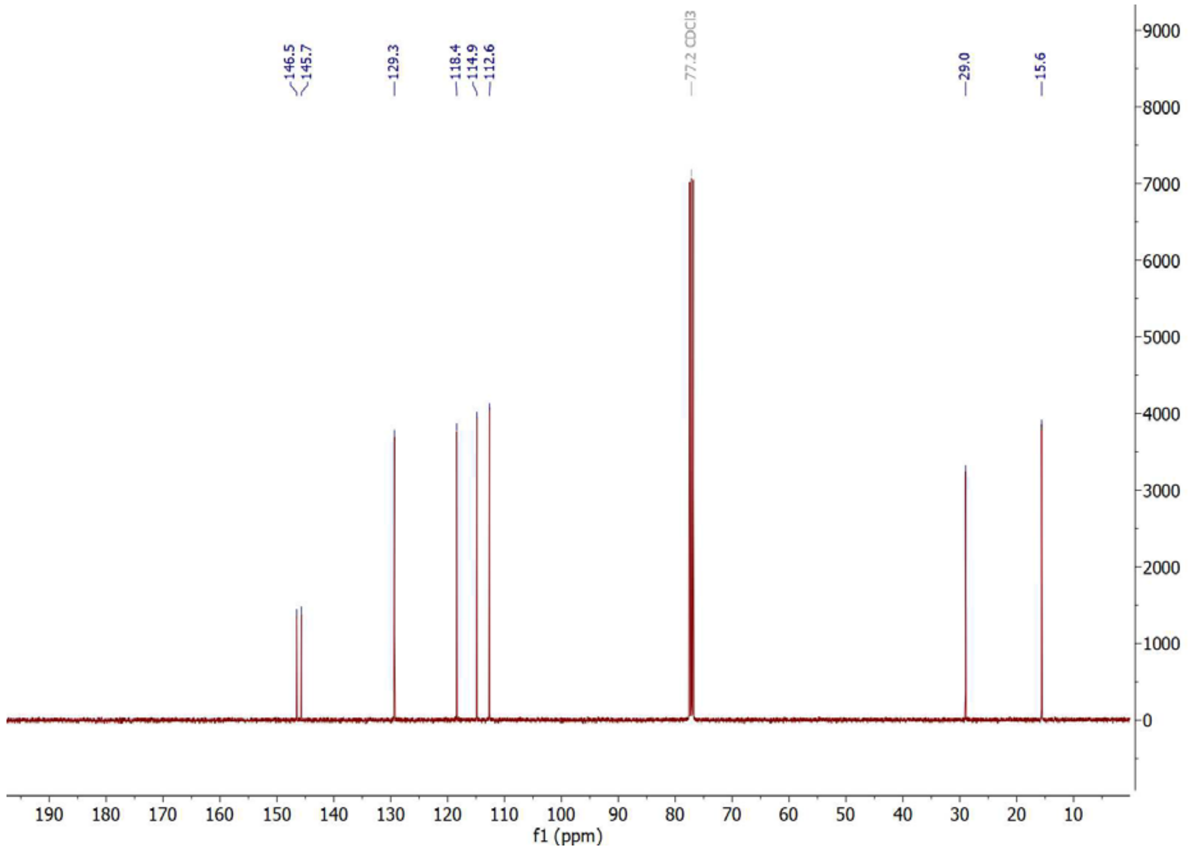
146.5
145.7
129.3
118.4
114.9
112.6
77.2 CDCl3
29.0
15.6
f1 (ppm)
